# Influenza Virus Reassortment Is Enhanced by Semi-infectious Particles but Can Be Suppressed by Defective Interfering Particles

**DOI:** 10.1371/journal.ppat.1005204

**Published:** 2015-10-06

**Authors:** Judith M. Fonville, Nicolle Marshall, Hui Tao, John Steel, Anice C. Lowen

**Affiliations:** 1 Center for Pathogen Evolution, Department of Zoology, University of Cambridge, United Kingdom; 2 Department of Microbiology and Immunology, Emory University School of Medicine, Atlanta, Georgia, United States of America; University of Michigan, UNITED STATES

## Abstract

A high particle to infectivity ratio is a feature common to many RNA viruses, with ~90–99% of particles unable to initiate a productive infection under low multiplicity conditions. A recent publication by Brooke et al. revealed that, for influenza A virus (IAV), a proportion of these seemingly non-infectious particles are in fact semi-infectious. Semi-infectious (SI) particles deliver an incomplete set of viral genes to the cell, and therefore cannot support a full cycle of replication unless complemented through co-infection. In addition to SI particles, IAV populations often contain defective-interfering (DI) particles, which actively interfere with production of infectious progeny. With the aim of understanding the significance to viral evolution of these incomplete particles, we tested the hypothesis that SI and DI particles promote diversification through reassortment. Our approach combined computational simulations with experimental determination of infection, co-infection and reassortment levels following co-inoculation of cultured cells with two distinct influenza A/Panama/2007/99 (H3N2)-based viruses. Computational results predicted enhanced reassortment at a given % infection or multiplicity of infection with increasing semi-infectious particle content. Comparison of experimental data to the model indicated that the likelihood that a given segment is missing varies among the segments and that most particles fail to deliver ≥1 segment. To verify the prediction that SI particles augment reassortment, we performed co-infections using viruses exposed to low dose UV. As expected, the introduction of semi-infectious particles with UV-induced lesions enhanced reassortment. In contrast to SI particles, inclusion of DI particles in modeled virus populations could not account for observed reassortment outcomes. DI particles were furthermore found experimentally to suppress detectable reassortment, relative to that seen with standard virus stocks, most likely by interfering with production of infectious progeny from co-infected cells. These data indicate that semi-infectious particles increase the rate of reassortment and may therefore accelerate adaptive evolution of IAV.

## Author Summary

Since the genome of an influenza A virus has eight non-contiguous segments, two influenza A viruses can exchange genes readily when they infect the same cell. This process of reassortment is important to the evolution of the virus and is one reason why this pathogen is constantly changing. It has long been known that a large proportion of the virus particles that influenza and many other RNA viruses produce are not fully infectious, but the biological significance of these particles has remained unclear. Here we show that virus particles that deliver incomplete genomes to the cell enhance the rate of reassortment. Thus, despite their limited potential to produce progeny viruses, these incomplete particles may play an important role in viral evolution.

## Introduction

The influenza A virus (IAV) genome comprises eight segments of negative sense RNA, each of which encode at least one essential viral protein [1,2]. This genome structure supports the generation of viral diversity through two major mechanisms: genetic drift due to an error prone viral polymerase, and exchange of gene segments between viruses through reassortment [3]. While drift allows the accumulation of small changes over time, reassortment allows substantial genetic change to occur quickly. Reassortment is highly prevalent among avian and swine IAVs and has been implicated repeatedly in the emergence of epidemically significant human strains [reviewed in 4]. The 1957, 1968 and 2009 pandemic strains arose through reassortment involving seasonal human strains and viruses adapted to avian and/or swine hosts [5–7]. In addition, reassortment among co-circulating human IAVs facilitated the spread worldwide of adamantane resistant H3N2 viruses and has brought about unusually severe seasonal epidemics including the Fujian-like outbreak in 2003/2004 [8–11]. The potential for reassortment to purge the viral genome of deleterious changes and bring together multiple beneficial mutations makes it a powerful catalyst of viral evolution [12].

The ratio of total particles to plaque forming units for influenza and other RNA viruses is on the order of 10:1 to 100:1 [13–17]. Thus, only ~1–10% of virions are thought to initiate productive infection of a cell under low multiplicity conditions of infection. The precise make up of the remaining virus particles is not clear but is likely a mixture of virions carrying qualitatively different defects [18,19]. Some may be non-infectious in that they fail to deliver viral RNA to the site of replication due to the lack of a genome, defects at the protein level, or a stochastic failure to initiate infection. Some may be classical defective-interfering (DI) particles, which carry one or more segments with a large internal deletion and act as parasites, hindering the production of fully infectious progeny [20–23]. Some will harbor a lethal point mutation in one or more segments [24,25]. Finally, some virions may be semi-infectious particles, which deliver fewer than eight segments to the nucleus [26,27]. Like DI particles and those with a lethal point mutation, SI particles cannot complete the viral life cycle. In contrast to these other particle types, however, SI virions do not carry a defective gene and are therefore not expected to interfere with the production of infectious progeny in the context of co-infection. Support for the existence of semi-infectious particles was recently gleaned through a careful analysis of viral protein expression in individual infected cells [27]. The majority of cells infected at low multiplicity failed to express one or more viral proteins, suggesting that the corresponding genes were disrupted or missing entirely from the viral genome. In that study, the probability of any segment being present and functional was estimated to be 0.781, which suggests that semi-infectious particles outnumber fully infectious particles by 6:1 [27].

In terms of their potential biological significance, there is an important difference between non-infectious and DI or SI particles. Any genetic material packaged into non-infectious particles will not be replicated. In contrast, under high multiplicity conditions, the genomes of DI and SI particles can be propagated through complementation by a co-infecting virus. This phenomenon of complementation, termed multiplicity reactivation, yields a greater number of infected and co-infected cells than would be predicted based on infectious titers determined at limiting dilution [28,29]. In addition, fully infectious viral progeny emerging from cells co-infected with only DI and/or SI viruses would necessarily be reassortant. Due to the anticipated increase in co-infected cells and the requirement for reassortment to yield fully infectious progeny from two incomplete parents, we hypothesized that the presence of DI or SI particles in an influenza virus population would promote genetic diversification through reassortment.

We tested this hypothesis using a combination of computational and experimental approaches. Our previously described system [30] for studying reassortment in the absence of fitness differences among parental and progeny strains was central to the experimental work and allowed the development of a relatively simple and robust model. Viral infection of cultured cells with two phenotypically identical viruses was simulated computationally at a range of multiplicities of infection. The model was then used to indicate expected relationships among infection, co-infection and reassortment in the absence and presence of increasing levels of semi-infectious particles. We tested the model by comparing co-infection with standard virus stocks to that with viruses that were UV irradiated to artificially increase SI particle content. By comparing experimental outcomes to the model, we were able to estimate semi-infectious particle content of non-irradiated virus stocks and obtained results in agreement with those of Brooke et al. indicating a high proportion of SI particles in IAV populations [27]. Our results furthermore suggest that the frequency with which each of the eight segments is missing from a virion varies among the segments. When the effect of DI segments was tested in the model, we found that their presence could promote or suppress reassortment relative to theoretical “perfect” virus stocks, depending on the potency with which a modeled DI segment interfered with infectious progeny production. To test the effect of DI particles experimentally, we used serial passage at high MOI to enrich for DI particles and studied co-infection with these virus stocks. The results indicated that DI segments reduce measured reassortment efficiency relative to standard virus stocks. Reassortment levels observed with DI-rich viruses were, however, higher than those predicted in the absence of any type of defective particle. In sum, we show herein that delivery of incomplete or defective genomes to target cells promotes reassortment by increasing the proportion of productively infected cells that are co-infected.

## Results

### Computational modeling of influenza virus infection, co-infection and reassortment

To determine the expected relationships between infection, co-infection and reassortment, we performed simulations where a 1:1 mixture of viruses of type A and B were randomly distributed over a computational set of cells (see Methods for details). Multiplicity of infection (MOI) was varied. For each MOI, we evaluated all cells and determined which were infected (defined as cells infected with A, B or A and B) and which were co-infected (with A and B). We calculated the % reassortment expected for each infected cell, taking into account the number of virions of each virus type present and allowing the segments to assort at random [30]. For example, cells that are infected with only a single virus type will produce 0% reassortant progeny, while cells infected with one of each type are expected to produce 99.22% reassortant progeny. The average % reassortment for all cells was then calculated to reflect the pool of progeny viruses released from all infected cells.

**Fig 1:**
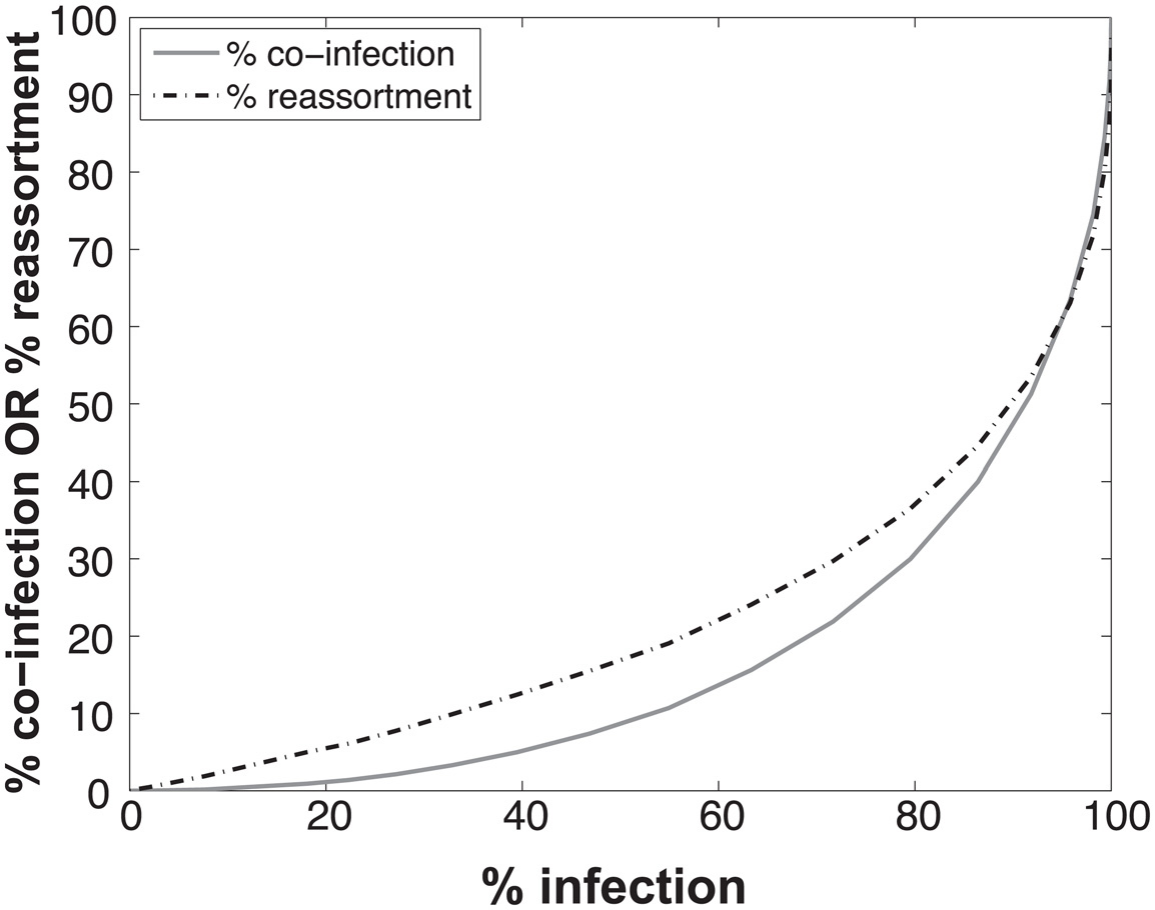
Relationships between % infection and % reassortment or % co-infection, as predicted by computational simulation of co-infection with viruses of two types. Viruses of type A and type B were assigned at random to a computational set of cells over a range of MOIs. Cells infected with any virus type were counted as infected and cells infected with both A and B types were counted as co-infected. The proportion of progeny viruses carrying a reassortant genome produced from each infected cell was calculated, taking into account the number type A and type B viruses present in each cell. The average % reassortment for all infected cells is plotted.

The results, shown in Fig 1, indicate that % co-infection and % reassortment depend in a non-linear, but monotonically increasing fashion, on the % infection. For low levels of infection, the likelihood of infections with multiple virus types is small, hence the % co-infection is low. To illustrate the interrelationship among % infection, % co-infection and % reassortment, we offer an example: at a low level of co-infection of 3.3%, where infection was 32.87%, the average expected % reassortment is calculated to be 9.899%. This result makes sense because ~10% of infected cells are co-infected and these co-infected cells are expected to produce nearly 100% reassortant viruses, while the infected cells that are not co-infected will produce only parental (type A or B) progeny.

### Impact on predicted co-infection and reassortment levels of introducing semi-infectious particles into the model

Because they deliver an incomplete genome to the site of replication, semi-infectious particles (SI particles) are expected to affect levels of reassortment. To evaluate how the relationships among reassortment, infection and co-infection are impacted by incomplete virions, we introduced SI particles into the simulated A and B virus populations and varied their prevalence using the parameter P_P_. The value assigned to P_P_ indicates the probability that a given segment is present in a virus particle. (Note that a segment that is not “present” could be physically missing from the particle or, alternatively, could fail to be delivered to the site of replication.) We initially assigned the same P_P_ value to all eight segments and explored a range of values between 0.3 (where the probability that a virion has all eight segments is 0.3^8^ = 6.5x10^-5^) and 1 (where all virions contain eight segments).

The presence of SI particles in a virus population gives rise to different types of infected cells: those that express HA and those that do not, and those that produce virus and those that do not. Thus, to allow meaningful discussion of the impact of SI particles on infection, co-infection and reassortment, we have generated the lexicon presented in Table 1. Within a simulated co-infection we are able to monitor all infected cells; that is, all cells into which a virus enters. For the purposes of comparing results of the simulation to those of the experimental co-infections described below, however, it is useful to also monitor cells that are expected to express HA protein on their surface. In the model, we defined an HA positive cell as an infected cell that has at least one copy of PB2, PB1, PA, NP and HA gene segments [31,32]. Given that some semi-infectious particles may lack one or more of these segments, a cell can be infected but be HA negative. To count as dually HA positive (i.e. expressing HAs of both type A and B viruses), a cell must have copies of both HA segments and at least one copy of PB2, PB1, PA and NP segments. Thus, a cell can be co-infected (at least one virus A and one virus B entered the cell), but only express one (or none) of the HA types, depending on the segments present in the type A and B viruses.

**Table 1:** Terminology used herein to describe different types of infected cells and virus particles.

Term	Definition
Infected cell	A cell into which any virus has entered
Co-infected cell	A cell into which at least one virus type A and at least one virus type B has entered
Singly infected cell	A cell into which only one type of virus (A or B) has entered
Productively infected cell	A cell that produces viral progeny
Productively co-infected cell	A cell that has genes of viruses type A and B in any combination and produces viral progeny
Productively singly infected cell	A cell which produces viral progeny of only one type (A or B)
HA positive cell	An infected cell that expresses the HA protein on its surface (in the model, such a cell must have at least one functional copy of each of PB2, PB1, PA, NP and HA)
Dually HA positive cell	A co-infected cell that expresses type A and type B HA proteins on its surface (in the model, such a cell must have at least one functional copy of each of PB2, PB1, PA, NP and HA_A_ and HA_B_)
Reassortant virus	A virus carrying genes of type A and type B in any combination
Multiplicity of infection (MOI)	The average number of virus particles that enter one cell
P_P_	The probability that a given segment is present in a virion and copied following infection. Based on their biological properties, segments with nonsense or frameshift mutations or with large internal deletions (DI segments) are treated as present.
P_I_	The probability that a segment which is present is also intact and functional
DIX	The factor by which a single DI segment delivered to a productively infected cell changes the output of infectious progeny from that cell.
Fully infectious virus	A virus that can initiate a productive infection in the absence of co-infection
Semi-infectious virus	A virus that delivers fewer than eight segments to the site of replication in the cell. An SI particle cannot complete the viral life cycle in the absence of complementation but does not interfere with infectious progeny production.
Defective-interfering virus	A virus that delivers one or more defective gene segments to a cell. A DI particle cannot complete the viral life cycle in the absence of complementation and interferes with the production of fully infectious progeny in the context of co-infection.

**Fig 2:**
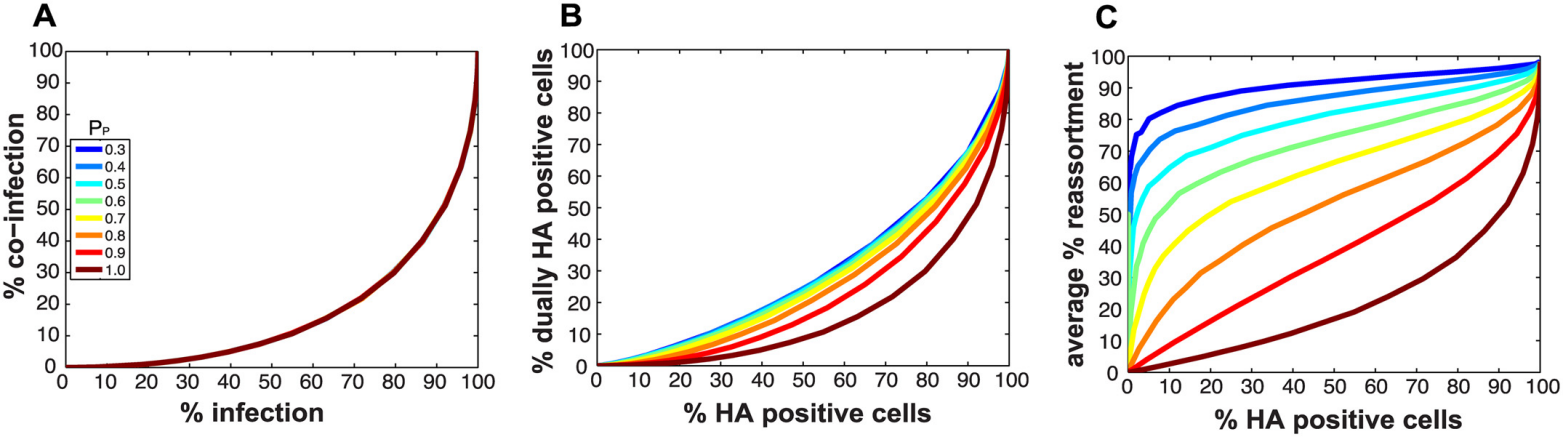
Impact on % infection, % co-infection, and % reassortment of introducing semi-infectious particles into the model. The probability that a given segment is present in each virus particle (P_P_) was varied from 1.0 to 0.3 within the model. The color scale indicates the value assigned to P_P_ in each simulation. A) The relationship between % infected and % co-infected cells is not affected by changing P_P_. B) The relationship between % HA positive cells and % dually HA positive cells varies with P_P_, due to the potential for segments that are required for HA gene expression to be missing from the infecting virus particle(s). C) The relationship between % HA positive cells and expected % reassortment, averaged across all productively infected cells, changes markedly with P_P_.

Fig 2A monitors the cells that were infected and co-infected, regardless of the presence of segments needed for HA expression, and shows that the results are insensitive to parameter P_P_. This outcome is as expected since the absence of segments does not alter infection status. However, when studying HA positive cells and dually HA positive cells, the results vary with P_P_ (Fig 2B). For a given % HA positive cells, the % dually HA positive cells increases as more viral genomes become incomplete (lower P_P_). This observation can be explained as follows. To achieve a given percentage of HA positive cells with more incomplete genomes, the number of virions per cell must increase to allow sufficient complementation. If the number of virions per cell (i.e. the MOI) is increased, the percentage of cells that are dually HA positive will also rise.

Similarly, missing segments will affect reassortment: a cell infected with one virion of type A and one virion of type B, both missing a different segment and thus complementing the other, will by definition produce 100% reassortant progeny. However, missing segments can also prevent progeny from being made, as at least one copy of each of the 8 segments is required to produce progeny in the model. In Fig 2C, reassortment levels expected under various conditions of P_P_ were calculated by averaging the expected % reassortment across all cells that were able to produce progeny. The results show that average % reassortment readily increases as P_P_ is lowered and more viral genomes are incomplete. This observation reflects the requirement for complementation, achieved via multiple infection, for infected cells to produce progeny viruses when viral populations are characterized by lower values of P_P_.

### Testing of the model: Co-infection and reassortment observed following co-inoculation of cells with standard virus stocks

**Fig 3:**
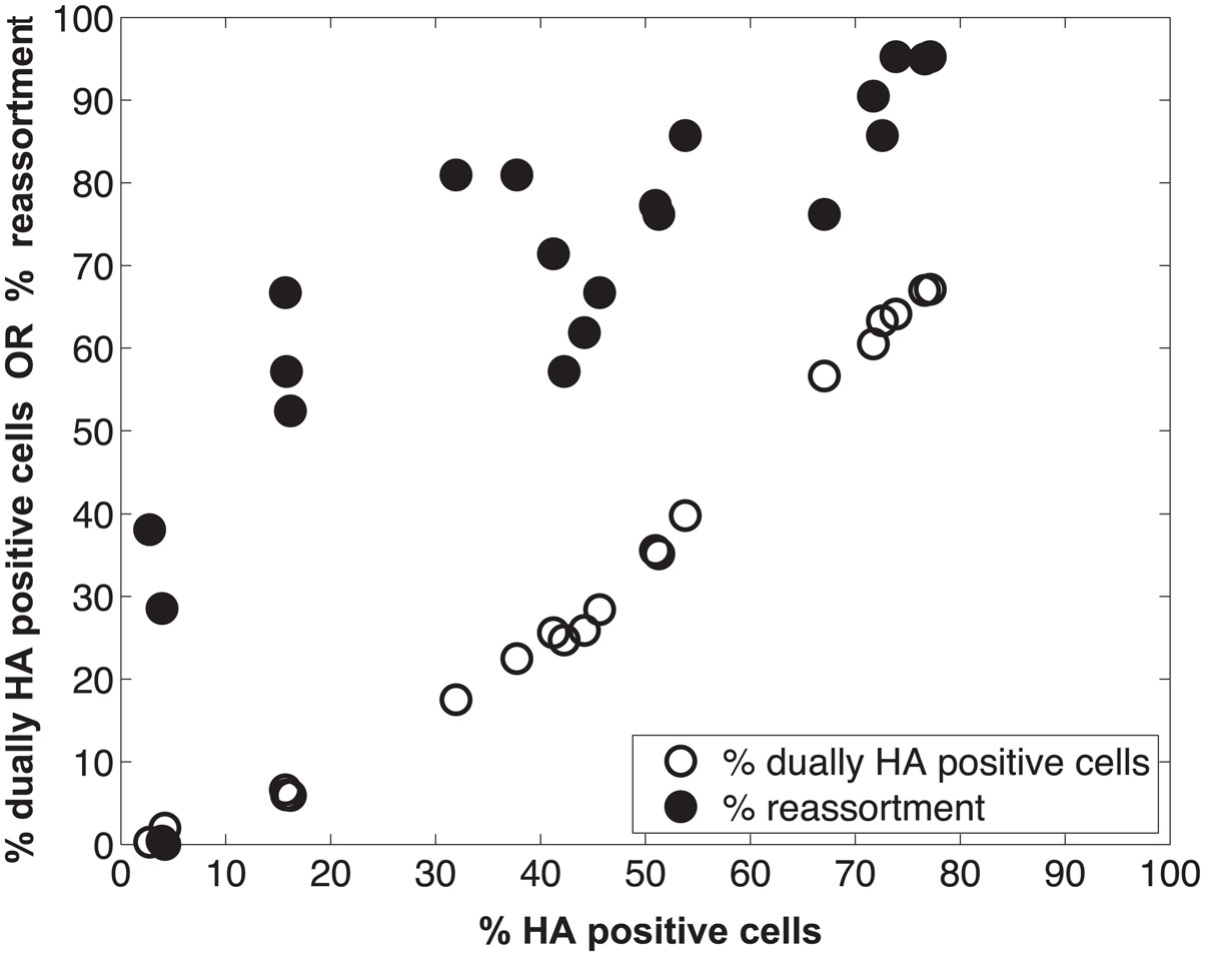
Measurement of HA positive cells, dually HA positive cells and reassortment following co-infection of MDCK cells with Pan/99wt and Pan/99var viruses. Pan/99wt and Pan/99var viruses were mixed in equal proportions and used to inoculate MDCK cells at a range of MOIs. Infection at each MOI was performed in triplicate. Following a single cycle of infection, cell culture supernatants were stored and HA positive and dually HA positive cells were identified by flow cytometry. Clonal viral isolates (n = 18–21) derived from each cell culture supernatant were genotyped by high resolution melt analysis to allow calculation of % reassortment. Individual data points, each corresponding to one cell culture dish, are plotted.

We performed a series of co-infections in Madin Darby canine kidney (MDCK) cells at a range of MOIs and monitored reassortment outcomes. To avoid fitness differences among parental and reassortant progeny viruses that could complicate the interpretation of results, we used our previously described A/Panama/2007/99 (H3N2) wild-type (Pan/99wt) and variant (Pan/99var) viruses. These viruses differ by silent mutations introduced into each gene segment of Pan/99var virus and by the insertion of a His epitope tag in the Pan/99wt virus vs. an HA tag in the Pan/99var virus [30]. Infections were performed in triplicate, synchronized by allowing virus attachment at 4°C and limited to a single cycle by the addition of ammonium chloride at 3 h post-infection [33]. At 12 h post-infection, supernatants were collected to genotype released virus and cells were processed for flow cytometry to enumerate cells with surface expression of the Pan/99wt and Pan/99var HA proteins (using the His and HA epitope tags). The resultant data were analysed by examining the relationship between i) % cells positive for any HA and % cells dually HA positive and ii) % cells positive for any HA and % reassortment (Fig 3). The results show that both % dually HA positive cells and % reassortment increase monotonically with % HA positive cells, but with differing patterns. The % dually HA positive cells shows a nearly linear relationship with % HA positive cells. In contrast, % reassortment increases quickly at lower levels of % HA positive cells, but plateaus at higher levels of % HA positive cells (Fig 3).

### Refinement of the model: Varying P_P_ among the segments was necessary to achieve a fit with the experimental data

**Fig 4:**
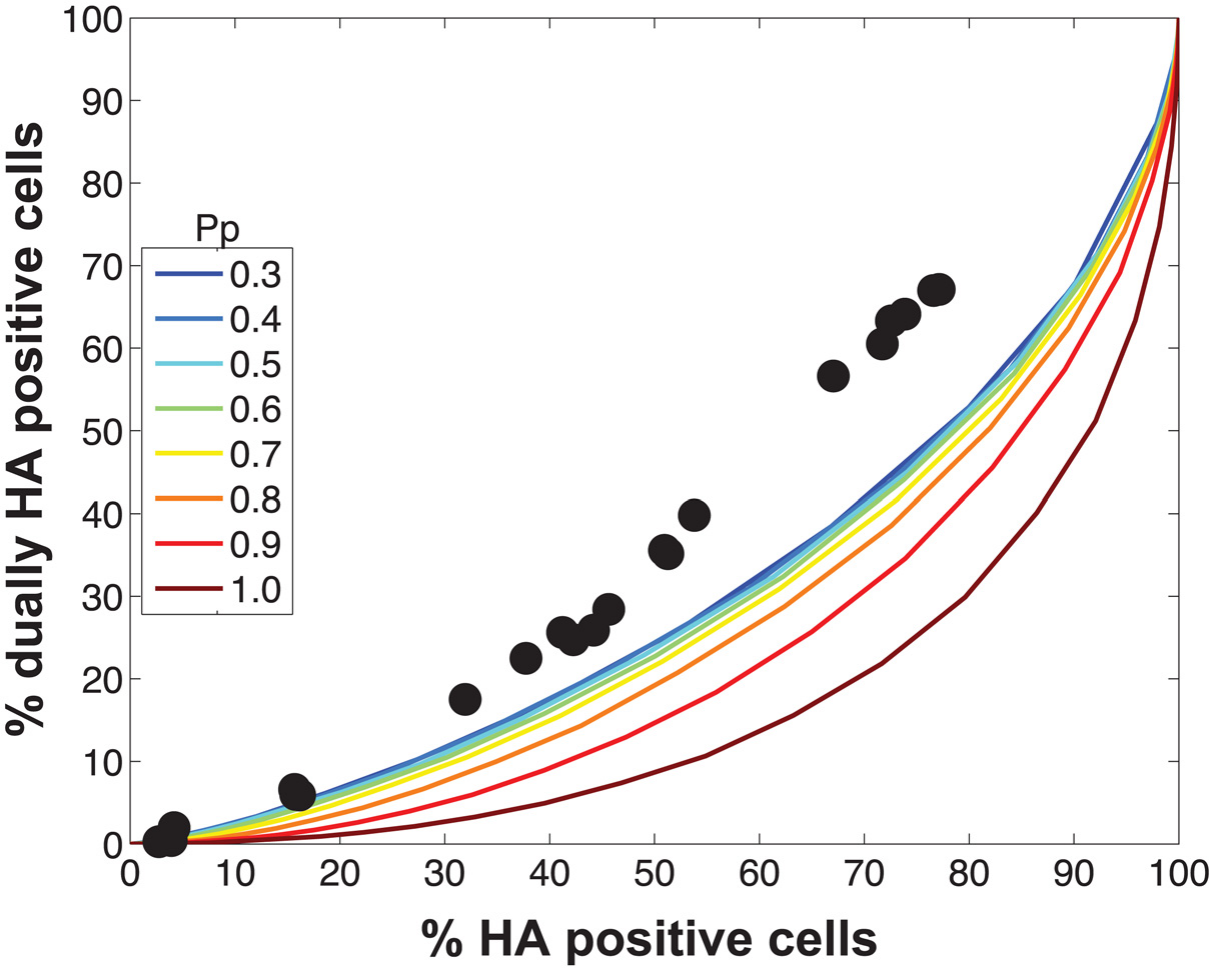
Modeled outcomes do not match the observed relationship between HA positive and dually HA positive cells when P_P_ is constant among the eight segments. Experimental data points, plotted with black circles, are overlaid on colored lines that indicate the predicted relationships between % HA positive cells and % dually HA positive cells given a range of P_P_ values. In this version of the model, the same P_P_ value was assigned to all eight segments. The color scale in the legend indicates the value assigned to P_P_ in each simulation.

**Fig 5:**
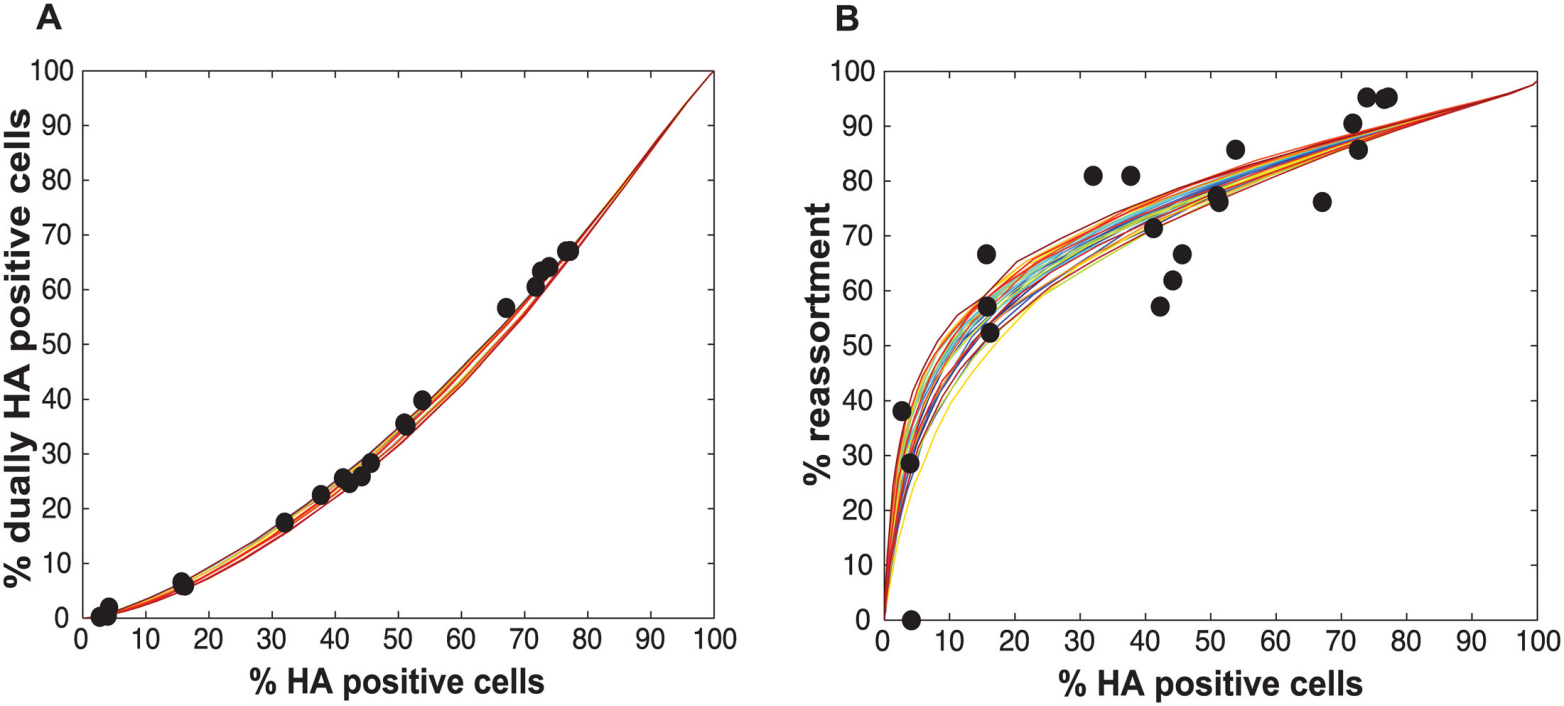
Varying P_P_ by segment yields good fit between modeled and observed relationships among HA positive cells, dually HA positive cells and reassortment. When P_P_ was allowed to vary among the segments (from 0.25 to 1.0 in increments of 0.25), several combinations of P_P_ values allowed a good fit between observational and modeled data. Shown here are the modeled results for the 1% of P_P_ combinations yielding the best fit (i.e. the top 28 of the 2800 settings tested). The 28 lines are colored from best (blue) to worst (red) fit. Experimental data points, plotted with black circles, are overlaid on these colored lines to allow comparison between experimental and modeled results for % HA positive cells vs. % dually HA positive cells (A) and % HA positive cells vs. % reassortment (B).

Initial comparisons of the experimental data with the model revealed a poor match for the relationship between % HA positive cells and % dually HA positive cells, regardless of the values assigned to P_P_ (Fig 4). We hypothesized that the assumption that P_P_ is constant among the eight segments might account for the discrepancy. We therefore modified the model to allow P_P_ to differ among the segments. Specifically, P_P_ was varied between 0.25 and 1.0 in increments of 0.25 and all possible combinations were tested, taking into account the redundancy of PB2, PB1, PA and NP as well as that of NA, M and NS in our readouts. A total of 2800 possible P_P_ combinations were evaluated. We quantified the fit for each of the 2800 combinations of P_P_ values as the sum of the distances between each experimental data point and the lines plotted using modeled results. This sum of errors was calculated for i) % HA positive cells vs. % dually HA positive cells; ii) % HA positive cells vs. % reassortment; and iii) % dually HA positive cells vs. % reassortment. The modeled results for the top 1% of P_P_ combinations are shown in Fig 5 together with the experimental data. These analyses highlighted that several P_P_ combinations gave results that matched the experimental data well. Given the uncertainty in the experimental measurements, the lines plotted in Fig 5 cannot be said to be meaningfully different. Additionally, the error between the experimental data and computational model varied slightly as a result of small stochastic variations in the outcome of the computational model when the same settings were repeated, resulting in small changes and uncertainties of the rank order of the top runs. Finally, note again that there are redundancies among certain segments (PB2, PB1, PA and NP are equivalent, as are NA, M and NS). Therefore, we do not consider the parameters giving the best fit with the data (as follows for segments 1–8, respectively: 0.25, 0.5, 0.75, 0.75, 1, 1, 1, 1), to be the final answer. Instead, we investigated which features the top 1% of P_P_ combinations had in common: each included at least one segment among PB2, PB1, PA and NP with P_P_ = 0.25; HA with P_P_ = 0.75; and at least 3 and up to 8 segments with P_P_<1.0. Taking the product of all eight P_P_ values yields the proportion of virions with all eight segments present, which for the best fit was 7.0%. When this proportion was determined from all sets of eight P_P_ values shown in Fig 5, the range obtained was 2.2–9.4%. In sum, comparison of the experimental data obtained with standard virus stocks to the model revealed that, for the model to fit the data, P_P_ must be less than 1.0 for multiple segments and P_P_ of the eight segments cannot be equivalent.

### Testing of the model: Predicted and observed impact of UV treated particles on co-infection and reassortment outcomes

**Fig 6:**
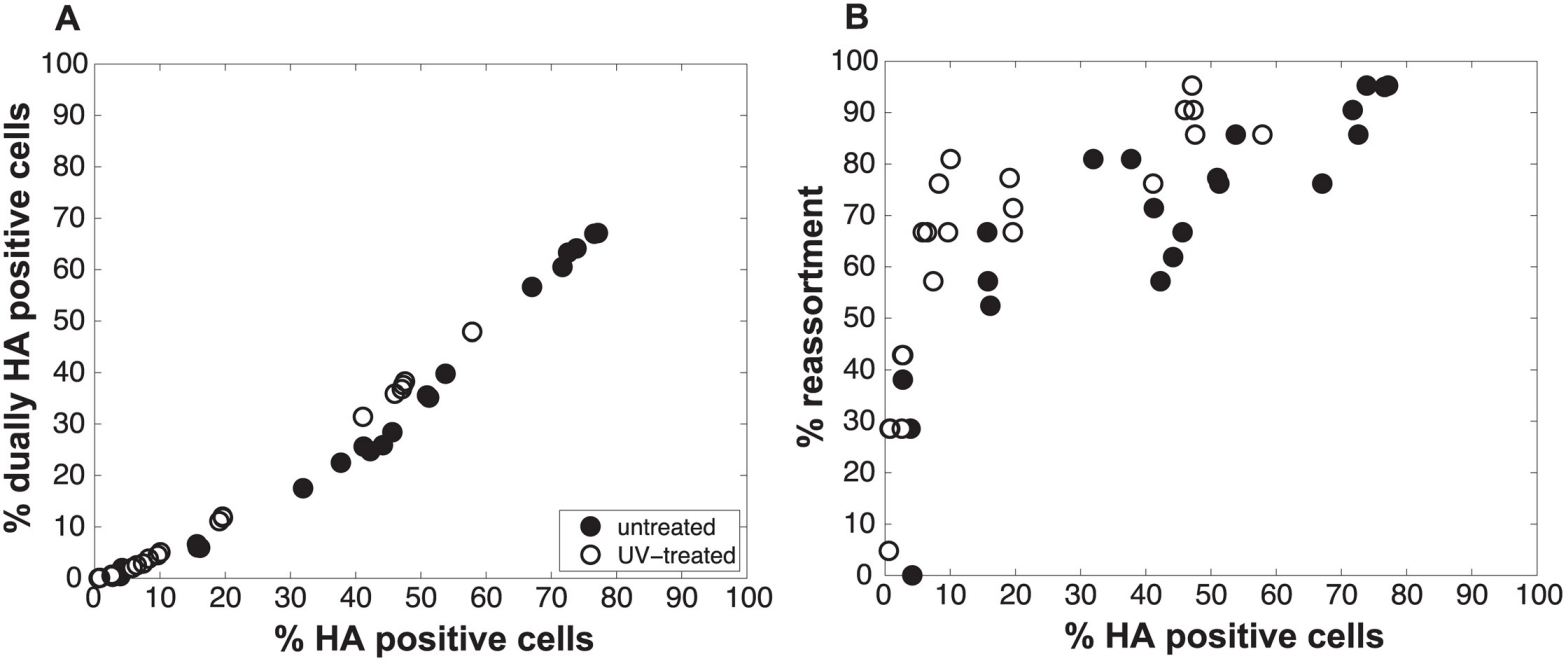
Increasing semi-infectious particle content by UV irradiation of virus stocks augments observed % reassortment. Pan/99wt and Pan/99var viruses were exposed to low dose UV light sufficient to reduce viral titers by ~10-fold (white circles) or were mock treated (black circles). Treated wt and var viruses were mixed in equal proportions and mock treated wt and var viruses were mixed in equal proportions. Each mixture was used to inoculate MDCK cells at a range of MOIs, in triplicate. Following a single cycle of infection, cell culture supernatants were stored and HA positive and dually HA positive cells were identified by flow cytometry. Clonal viral isolates (n = 18–21) derived from each cell culture supernatant were genotyped by high resolution melt analysis to allow calculation of % reassortment. A) Relationship between % HA positive cells and % dually HA positive cells. B) Relationship between % HA positive cells and % reassortment. Individual data points, each corresponding to one cell culture dish, are plotted. Data from mock treated samples (black circles) are also presented in Fig 3 and are included here to allow direct comparison with data from UV treated samples.

To test the validity of our model and more rigorously evaluate the hypothesis that semi-infectious particles augment reassortment, we generated Pan/99wt and Pan/99var virus populations with increased semi-infectious particle content. This increase was achieved by exposing each virus to a low dose of UV irradiation. Since polymerase read-through of pyrimidine dimers is not possible, viral segments carrying UV lesions will not be replicated or transcribed and will behave similarly to missing segments. A UV dose sufficient to decrease PFU titers by approximately 10-fold was used. Co-inoculation of MDCK cells with Pan/99wt and Pan/99var viruses that had been UV treated was then performed at a range of MOIs (in parallel with co-infections using standard virus stocks, described above). Again, results were analysed by assessing the relationships among % HA positive cells, % dually HA positive cells and % reassortment. As predicted by the model, co-inoculation with UV treated viruses yielded similar levels of dually HA positive cells, but higher reassortment frequencies at intermediate levels of HA positivity, compared to co-inoculation with mock treated viruses (Fig 6). To evaluate whether UV treatment has a statistically significant impact on % reassortment, we performed a multiple linear regression analysis of % reassortment vs. log_10_ (% HA positive), treating UV as a categorical variable. The results showed that, for every increase of 1 log_10_ (% HA positive), the % reassortment goes up by 40% (P = 3.9x10^-16^). Having UV treatment further increases % reassortment by 16%, and is a significant categorical variable (P = 6.6x10^-6^). These data support the validity of the model and specifically verify the model’s prediction that increasing semi-infectious particles in a virus population enhances the production of reassortant progeny.

**Fig 7:**
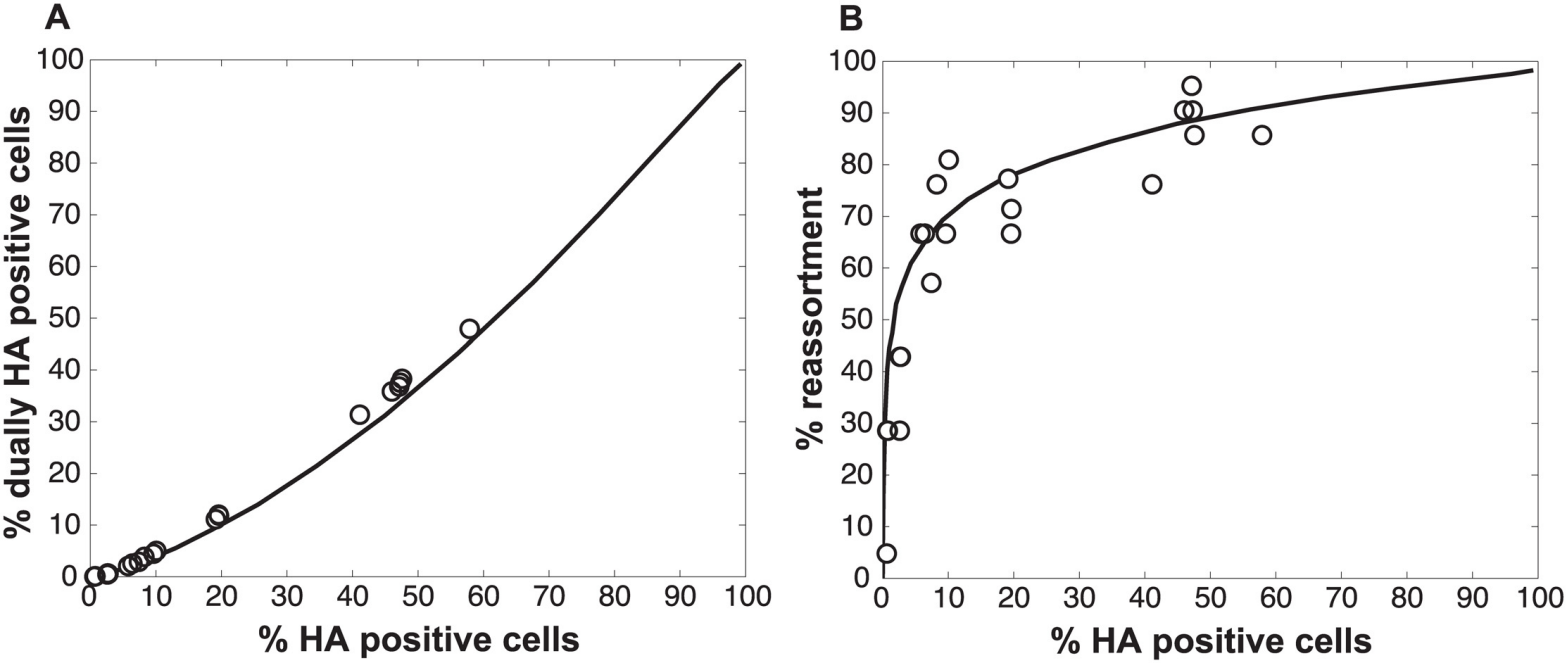
Results observed with UV treated Pan/99wt and Pan/99var viruses match simulated co-infections in which each virus carries an average of 2.0 UV hits per genome. Experimental data points obtained with UV treated virus stocks, plotted with open circles, are overlaid on a black line indicating the model’s predicted relationships for % HA positive cells vs. % dually HA positive cells (A) and % HA positive cells vs. % reassortment (B). Computational viruses carried an average of 2.0 UV hits per genome when P_P_ settings found to best match the data obtained with untreated virus stocks were used (0.25, 0.5, 075, 0.75, 1, 1, 1, 1 for segments 1–8, respectively).

We also assessed whether the increase in reassortment seen with UV treatment was quantitatively related to the observed difference in infectivity between UV treated and untreated virus stocks. Analysis of results with the UV treated virus stocks indicated that the levels of reassortment and co-infection observed best matched those predicted for a virus population that had suffered 2.0 hits per genome on average (Fig 7). Based on a Poisson distribution of UV hits per virus, this UV dose would be expected to reduce PFU titer by 7.4-fold. The observed knock-down in PFU titers with UV treatment was 11-fold. These results are comparable, particularly when one considers the typical range of error of a plaque assay (approximately 2-fold) [34], and therefore further support the validity of the model.

### Deciphering the mechanism by which SI particles promote reassortment

**Fig 8:**
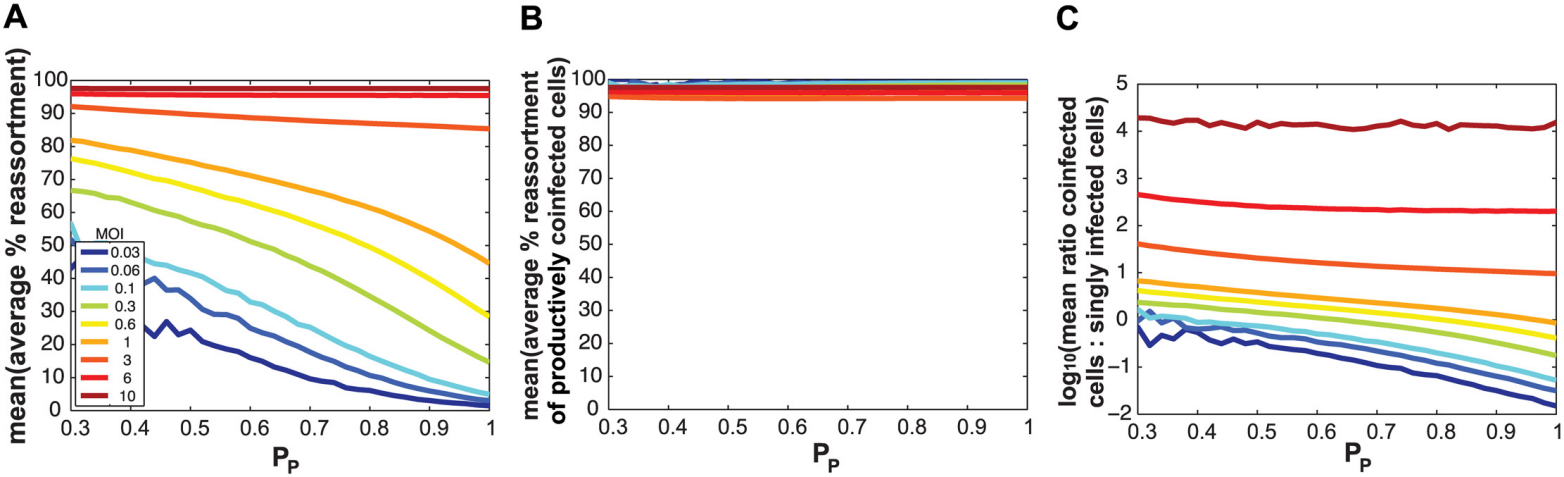
Semi-infectious particles increase reassortment at a given MOI by increasing the proportion of infected cells that are co-infected. Results of computationally simulated co-infections are shown. In each simulation, multiplicity of infection in terms of total particles/ cell was held constant and is indicated by the color scale inset in panel (A). The same P_P_ value, plotted on the x-axis, was assigned to all eight segments and was varied from 0.3 to 1.0 in increments of 0.1. Twenty replicates were run, and the mean of these 20 runs is shown. In (A), the average % reassortment expected under each condition is plotted. Fully infectious progeny viruses generated from all infected cells are considered in this analysis. The results show that, at each MOI, the % of viruses that are reassortant increases with decreasing P_P_. In (B), the average % reassortment for productively co-infected cells is plotted for each MOI condition. Here, only those fully infectious viruses generated in productively co-infected cells are considered. The results show that, regardless of MOI and P_P_, the vast majority of fully infectious viruses emerging from co-infected cells have a reassortant genotype. In (C), the ratio of co-infected cells to singly infected cells is plotted on a log 10 scale. This plot shows that, at MOIs less than 3 particles/cell, this ratio increases with decreasing P_P_. In other words, where P_P_ is lower, a greater proportion of cells are potential vessels for reassortment. A log 10 scale was used to allow visualization of all lines on one graph.

We hypothesized that SI particles could augment reassortment through one of three, non-mutually exclusive, mechanisms: i) by simply increasing the number of particles entering each cell (i.e. the MOI) given a constant number of productively infected cells; ii) by increasing the frequency of reassortant viruses emerging from productively co-infected cells at a given MOI; and/or iii) by increasing the proportion of productively infected cells that are co-infected at a given MOI. To distinguish among these possibilities, we used the model to examine the impact of varying P_P_ *and* MOI on levels of reassortment and co-infection. To simplify this theoretical analysis, we assigned the same P_P_ value to all segments. When MOI was held constant and % reassortment, averaged across all productively infected cells, was plotted as a function of P_P_, the result clearly showed increasing reassortment with declining P_P_ (Fig 8a). This result indicates that SI particles do not act on reassortment solely by increasing MOI (item i above). When MOI was held constant and average % reassortment for only productively co-infected cells was analysed as a function of P_P_, the model indicated that levels of reassortment were high across the full range of P_P_ (Fig 8b). This result reflects the high efficiency of IAV reassortment in co-infected cells and excludes item ii above as an important mechanism driving enhanced reassortment with increasing SI content. Lastly, when MOI was held constant and the ratio of productively co-infected to productively singly infected cells was plotted as a function of P_P_, the results indicated that decreasing P_P_ leads to an increase in the proportion of cells that are potential vessels for reassortment (Fig 8c; note the log scale on the Y-axis). This last result reveals that mechanism iii above is functional: addition of SI particles to a virus population increases the likelihood that productively infected cells will produce reassortant viruses by changing this population of cells to be more often co-infected, even when the number of virus particles is not changed.

### Modeling the impact of defective interfering particles on co-infection and reassortment outcomes

We undertook analysis of the impact of DI particles on influenza virus reassortment to confirm that the semi-infectious particles detected in our previous analyses were not, in fact, DI particles, and to determine the potential for these naturally occurring deletion mutants to contribute to viral evolution. Similar to SI particles, DI particles deliver an incomplete genome to the site of replication. DI particles differ from SI particles, however, in that they carry one or more segments with a large internal deletion [35–38]. Importantly, these internally deleted segments have been shown to interfere with the production of infectious progeny and to accumulate over multiple rounds of replication so that they quickly outnumber the corresponding standard genome segments [22,39–43]. Thus, DI particles are expected to affect levels of reassortment in two ways: by delivering an incomplete genome and by interfering with production of infectious progeny. To evaluate how the relationships among reassortment, infection and co-infection are impacted by DI virions, we introduced DI particles into the simulated A and B virus populations and varied their prevalence using the parameter P_I_. The value assigned to P_I_ indicates the probability that a given segment in a virus particle is intact (i.e. does not have an internal deletion or other lethal mutation). Since the polymerase segments of DI particles are more commonly found to be defective than the remaining five segments, we assigned P_I_ values < 1.0 to PB2, PB1 and PA, while maintaining P_I_ = 1.0 for HA, NP, NA, M and NS segments. The interfering behavior of defective segments was controlled with the parameter DIX, the fold change in infectious progeny production attributed to each single DI segment in a productively infected cell. When DIX = 0.5, a DI segment and the corresponding standard segment have equivalent likelihoods of being incorporated into progeny virions and thus half of the progeny produced will be non-infectious (carrying the DI) while half will be infectious (carrying the standard segment). Since the total number of virus particles produced by a given cell is held constant, a DI with DIX = 0.5 reduces infectious progeny by half. To account for the experimental observation that DI particles accumulate over multiple passages [23,41–44], we reasoned that DIX must be less than 0.5. The true value of DIX for a given DI segment is not, however, clear from the literature, may be variable depending on the context, and may vary among differing DI segments. In our analyses, we therefore varied DIX over a range of 0.05 to 0.5 or evaluated three disparate settings of 0.01, 0.1 and 0.45. With the aim of evaluating whether DI segments (rather than missing segments) could account for the reassortment outcomes shown in Fig 3, our initial analysis was performed with P_P_ = 1.0 for all segments.

**Fig 9:**
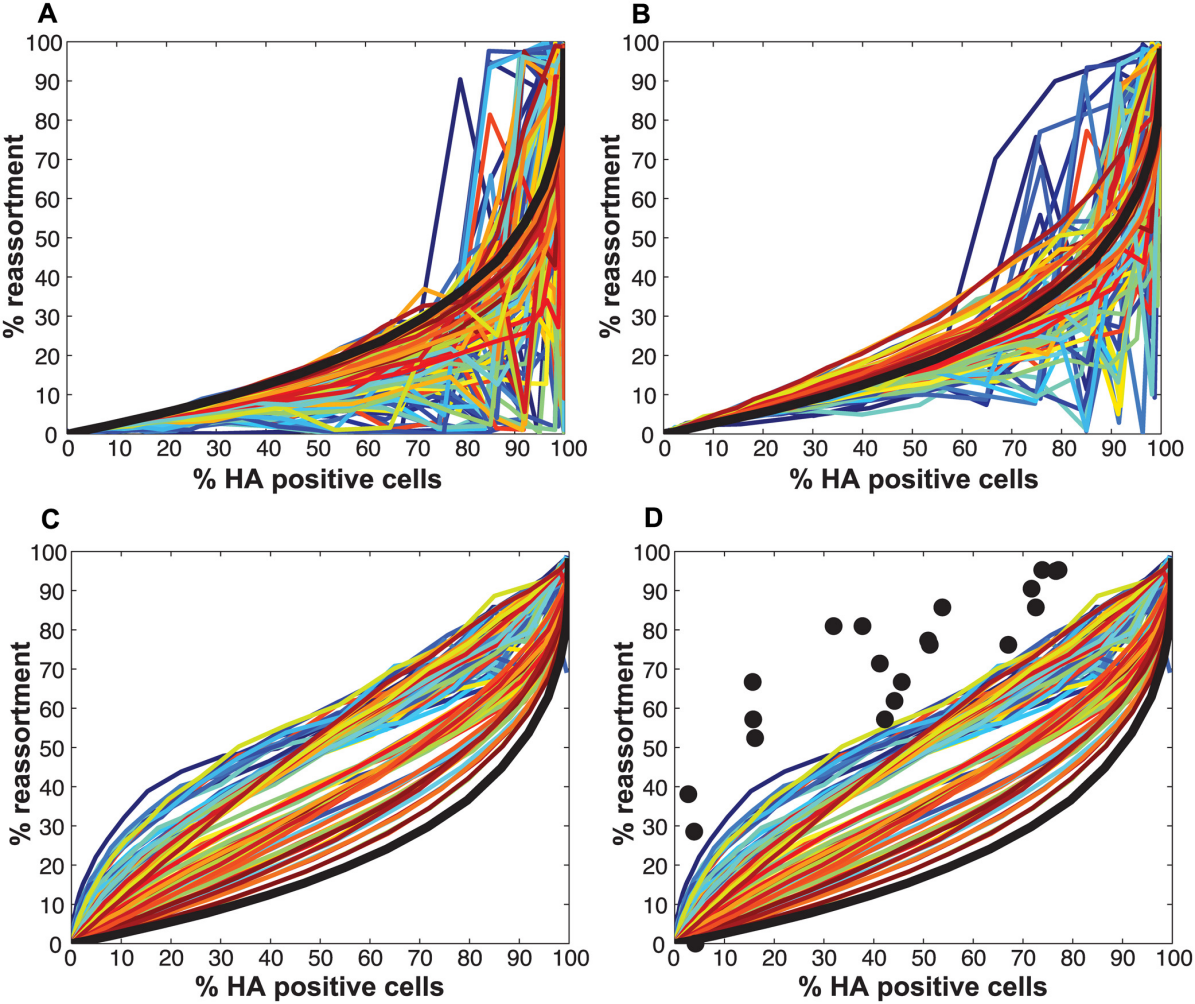
Impact on % reassortment of introducing defective interfering particles into the model. DI particles were introduced into the model by varying P_I_ values of PB2, PB1 and PA independently from 0.2–1.0 in increments of 0.2. Thus, 125 different settings for the prevalence of DI segments were tested. Computational virus populations A and B were assigned the same P_I_ values in each simulation. For each of the 125 settings, the potency with which the DI segments interfere with infectious virus production, DIX, was set to 0.01 (A), 0.1 (B) and 0.45 (C and D). In each panel, the color assigned to each of the 125 P_I_ settings reflects the product of P_I(PB2)_, P_I(PB1)_ and P_I(PA)_, with the highest product (0.75) in red and the lowest product (0.008) in blue. In addition, the line representing results obtained with P_I_ = 1.0 for all segments is shown in black for reference. (D) Experimental data obtained with standard virus stocks are plotted with black circles and overlaid on the modeled results for DIX = 0.45. This comparison indicates that, although the presence of mildly interfering DI segments enhances reassortment, this effect is not sufficient to account for the levels of reassortment observed with Pan/99wt and Pan/99var viruses.

The results of this computational analysis are shown in Fig 9. The results obtained for P_I_ values of 0.25–1.0, independently varied among PB2, PB1 and PA in increments of 0.25, are displayed, with DIX set to 0.01 (very potently interfering), 0.1 (potently interfering) and 0.45 (mildly interfering) in panels A, B and C, respectively. Note that stochastic effects occur in the simulations at low values of P_I_ and/or P_P_ due to a low “n” of computational cells producing the computational virus that is analyzed. These stochastic effects give rise to the noisy peaks seen in Fig 9 and later figures. The results with DIX of 0.01 reveal that very potently interfering DI segments are expected to suppress the production of infectious reassortant progeny viruses. When DIX was set to a more moderate value of 0.1, predicted levels of reassortment fell either above or below those for virus stocks in which all particles are complete, depending on the P_I_ values used. In contrast, regardless of P_I_, virus stocks carrying mildly interfering DI segments (represented with DIX = 0.45) were predicted to result in higher reassortment levels than virus stocks with only complete genomes. To test whether the reassortment outcomes observed experimentally with Pan/99wt and Pan/99var standard virus stocks could be accounted for by the presence of mildly interfering DI segments, we overlaid the experimental data with the modeled predictions for DIX = 0.45 and the broad range of P_I_ value combinations analyzed previously (Fig 9D). All conditions tested yielded % reassortment values lower than those seen experimentally. This result indicates that DI particles do not underlie the relatively high levels of reassortment observed with our standard virus stocks, and furthermore provides a theoretical prediction that DI particles will suppress reassortment relative to standard virus populations. We also evaluated a potential role for defective segments that carry a lethal point mutation rather than a deletion. Such mutations can arise in all eight segments and do not confer a competitive advantage upon the segment carrying them [40]. We therefore varied P_I_ for all eight segments in the model and assigned DIX = 0.5. These parameters also did not allow a good match between modeled and experimental results (S1 Fig). Thus, the presence of defective segments is not sufficient to explain the levels of reassortment as a function of % HA positive cells seen in Pan/99wt and Pan/99var virus co-infection.

### Testing of the model: Observed impact of defective interfering particles on co-infection and reassortment outcomes

**Fig 10:**
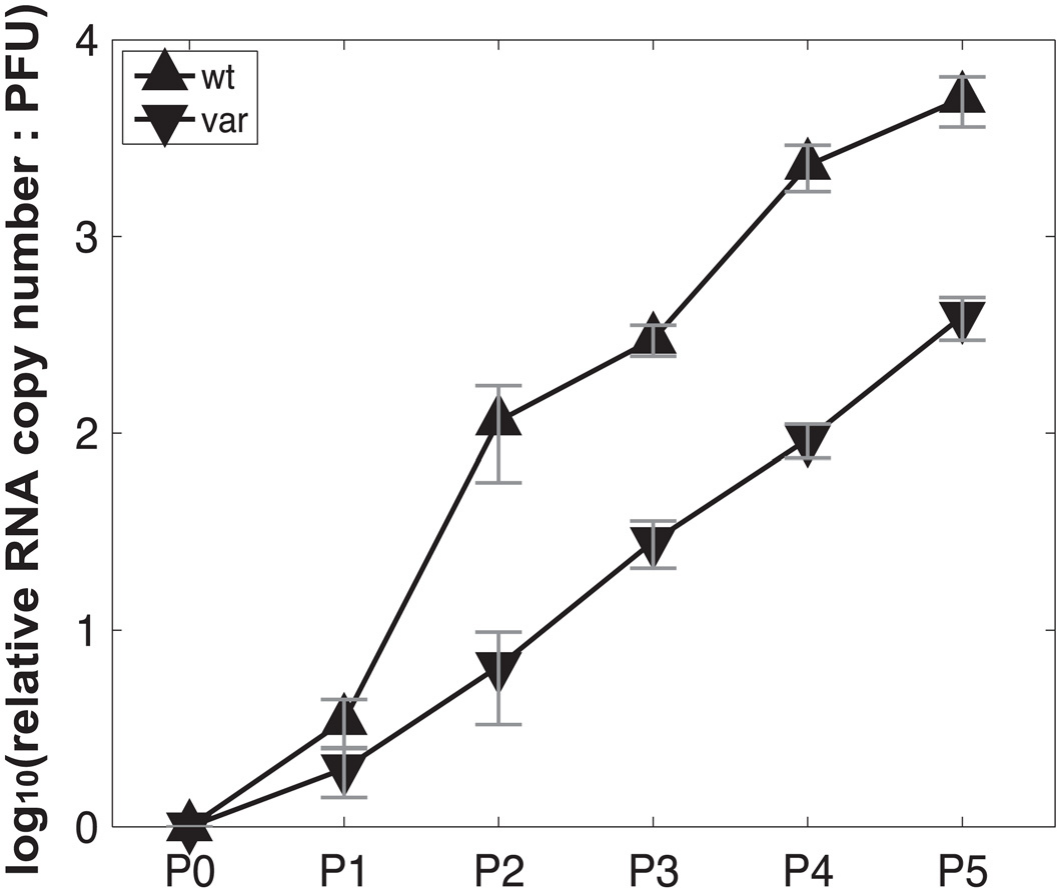
Production of virus stocks with high levels of DI particles: ratio of RNA copy number to PFU increases with serial passage at high MOI. Pan/99wt and Pan/99var viruses were passaged five times in MDCK cells. The first passage was performed at an MOI = 5 PFU/cell and subsequent passages were performed with undiluted cell culture supernatants. RNA copy numbers relative to egg-grown progenitor (“P0”) stocks were determined based on the average of NS, M and NA segments. Plaque assays were performed in triplicate. Error bars indicate standard deviation.

To test the model of IAV reassortment in the presence of DI particles, and differentiate among the outcomes predicted for differing values of DIX, we evaluated reassortment and HA positivity following co-infection with virus stocks that carried high levels of DI particles. Virus stocks rich in DI particles were generated by serial passage of Pan/99wt and Pan/99var viruses at high MOI in MDCK cells. As expected, an increase in the ratio of genome copy number to PFU, relative to the standard virus stocks, was observed with increasing passage number (Fig 10). We selected passage 3 (P3) and P4 virus stocks for further experiments since both wt and var viruses at these passage numbers showed >10-fold increases in the ratio of genome copy number to PFU, while the very low titers of the P5 viruses precluded their use. To confirm the presence of DI segments, we used an RT qPCR assay in which RNA copy number (relative to standard “P0” stocks) detected with primers binding near the 3’ end of the vRNA was compared to that obtained with primers binding internally, in a region typically deleted within DI segments [37,38]. The proportion of viral gene segments that were intact (P_I_), relative to P0 stocks, was calculated as described in the Methods and is reported in Table 2. The results reveal low P_I_ values for PB2, PB1 and/or PA segments of the P3 and P4 viruses, confirming that high proportions of these segments carried internal deletions (Table 2).

**Table 2:** Proportion of polymerase segments intact in P3 and P4 viruses, relative to P0 viruses.

	P_I_ (95% confidence interval)			
Segment	P3wt	P3var	P4wt	P4var
PB2	0.107 (0.027)	0.653 (0.31)	0.127 (0.078)	0.792 (0.30)
PB1	0.116 (0.016)	0.085 (0.021)	0.060 (0.012)	0.045 (0.011)
PA	0.301 (0.062)	0.634 (0.13)	0.203 (0.053)	0.395 (0.19)

**Fig 11:**
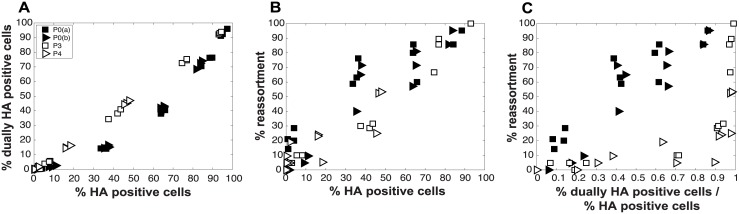
Virus populations dominated by DI particles give rise to a higher proportion of dually HA positive cells but a lower proportion of reassortant progeny viruses compared to virus populations with low DI content. Pan/99wt and Pan/99var viruses with matched passage histories were mixed in equal proportions (P0wt + P0var, P3wt + P3var and P4wt + P4var). Each mixture was used to inoculate MDCK cells at a range of MOIs, in triplicate. MOIs of P3 and P4 virus infections were calculated based on RNA equivalents relative to P0, such that comparable numbers of particles/cell were used in each case. A) Relationship between % HA positive cells and % dually HA positive cells. B) Relationship between % HA positive cells and % reassortment. C) Relationship between the proportion of HA positive cells that are dually HA positive and % reassortment. Results from two independent experiments are shown: data labeled as P0a were obtained in parallel with those for P3 and data labeled as P0b were obtained in parallel with those for P4.

To evaluate the consequences of DI particles for co-infection and reassortment frequencies, we co-inoculated MDCK cells with standard Pan/99wt (P0wt) and Pan/99var (P0var) viruses, P3wt and P3var viruses, or P4wt and P4var viruses at a range of MOIs. To allow comparison among P0, P3 and P4 viruses on a per particle level, multiplicities of infection were based on RNA copy number of the three shortest segments (NS, M and NA) rather than infectious titers. Infections were performed in triplicate and synchronized by allowing virus attachment at 4°C. At 12 h post-infection, supernatants were collected to genotype released virus and cells were processed for flow cytometry to enumerate Pan/99wt and Pan/99var infected cells. Trypsin was excluded but, in contrast to the infections described above, we did not add ammonium chloride at 3 h post-infection. Data were analyzed by examining the relationships between i) % HA positive cells and % HA dually positive cells, ii) % HA positive cells and % reassortment and iii) the proportion of HA positive cells that were dually HA positive and % reassortment (Fig 11). The results show enhanced frequencies of dually HA positive cells at a given % HA positive for the P3 and P4 virus stocks relative to P0 stocks (Fig 11A). This observation likely reflects the need for complementation to support the expression of an HA gene carried by a DI particle. Despite the occurrence of such complementation and detection of abundant dually HA positive cells, relatively few reassortant progeny viruses emerged from P3 and P4 virus co-infections (Fig 11B). This result is very clear when % reassortment is plotted against the proportion of HA positive cells that were dually HA positive (Fig 11C). Even when a high proportion of cells (up to 0.9) were dually HA positive and therefore infected with both wt and var viruses, co-infection with the P3 or P4 viruses stocks yielded <25% reassortment. As suggested by the model, this reduction in reassortant progeny relative to that produced by the P0 viruses is likely due to the interfering effects of short, DI, segments [45]. In other words, the predominance of progeny viruses with a parental genotype suggests that cells within the P3 or P4 co-infected dishes that are singly infected with a fully infectious virus produce the majority of the infectious progeny, even when such cells represent a small proportion of HA positive cells (Fig 11C). In sum, these data demonstrate that, as predicted by the model, the DI rich P3 and P4 virus stocks gave rise to fewer reassortant viruses compared to standard virus stocks.

### Comparison of experimental and theoretical reassortment outcomes suggests that P_P_ ≈ 1.0 for DI rich virus stocks

**Fig 12:**
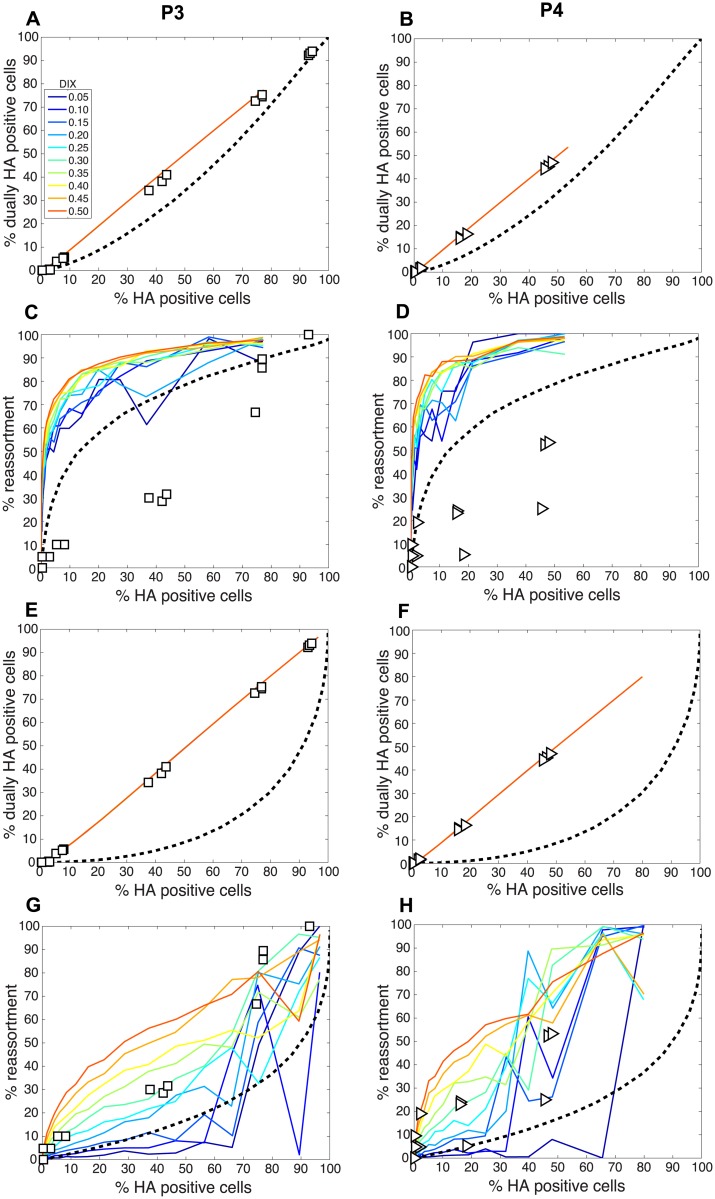
Comparison of modeled and experimentally determined relationships among % HA positive cells, % dually HA positive cells and % reassortment in the presence of DI particles. To allow comparison between experimental and theoretical results obtained with DI particles in the virus populations, P_I_ parameters measured for the P3 (panels A, C, E and G) and P4 (panels B, D, F and H) virus stocks were input into the model. DIX was varied from 0.05 to 0.5 in increments of 0.05 and is shown with a color scale in each panel, where blue represents DIX = 0.05 and orange is DIX = 0.5. In panels A-D, P_P_ values of 0.25, 0.5, 0.75, 0.75, 1.0, 1.0, 1.0, 1.0 for segments 1–8, respectively, were used. In panels E-G, P_P_ was set to 1.0 for all eight segments. A dashed line representing modelled results in the absence of DI particles (P_I_ = 1.0) is plotted as a reference.

We next compared the experimental results obtained with the P3 and P4 virus stocks to the model directly by overlaying the observed data points with the modeled predictions for % HA positive cells vs. % reassortment and % HA positive cells vs. % dually HA positive cells. In each case, P_I_ parameters measured for the P3 and P4 viruses stocks (Table 2) were used and DIX was varied from 0.05 to 0.5 in increments of 0.05. Since our results indicated that P_P_ was less than 1.0 for the standard Pan/99wt and Pan/99var virus stocks, we set P_P_ within the model to those values found above to yield the best fit between modeled and experimental data (Fig 5). The results, shown in Fig 12A-12D, indicated that, when combined with P_P_ values of 0.25, 0.5, 0.75, 0.75, 1.0, 1.0, 1.0, 1.0 for segments 1–8, respectively, the presence of DI particles at levels seen in the P3 and P4 virus stocks is expected to yield very high % reassortment across nearly all levels of % HA positive cells. In other words, when parameterized in this way, the model did not match the data. We therefore evaluated the outcomes when P_P_ was set to 1.0 for all segments and P_I_ to those measured for P3 and P4 virus stocks (Fig 12E-12H). Although the model gave a range of predictions depending on the value assigned to DIX, the results obtained with P_P_ = 1.0 (or P_P_ = 0.9; S2 Fig) for the DI-containing viruses were consistent with those observed following P3 and P4 virus co-infections. One explanation for this result is that short, DI, segments may be packaged (or delivered to the site of infection) more efficiently than standard segments.

To further explore the inter-relationships among P_P_, P_I_ and DIX, we tested a range of theoretical settings for each parameter within the model and show 12 representative results for % HA positive cells vs. % reassortment in S3 Fig. The theoretical outcomes displayed indicate that there is a complex interplay among P_P_, P_I_ and DIX in determining reassortment levels. Importantly, the presence of mildly interfering DI segments (i.e. those with DIX near to 0.5) in virus populations is predicted to enhance reassortment under all P_P_ conditions tested. This result indicates that a combined effect of missing segments and mildly interfering DI segments could lead to reassortment levels comparable to those observed experimentally with our standard Pan/99wt and Pan/99var virus stocks. As shown in Fig 9D, however, inclusion of DI segments but not missing segments in the theoretical virus populations is insufficient to account for the experimental reassortment data.

## Discussion

The results of both simulated and experimental IAV co-infections indicate that the presence of incomplete particles in parental virus populations enhances the frequency with which reassortant progeny viruses emerge. Our results suggest that both non-interfering, semi-infectious particles and classical defective interfering particles can act to enhance reassortment above that expected for theoretical virus populations that carry only intact genomes. The extent to which DI particles can enhance reassortment is limited, however, by the interference with infectious progeny production imposed by DI segments. As a consequence of this interference, DI particles suppress reassortment relative to that seen with standard, biological, virus stocks.

Higher average reassortment levels seen with SI particles are not due to increased efficiency of reassortment within individual co-infected cells, but rather are brought about by an increase in the proportion of productively infected cells that are co-infected when SI particles are present. This mechanism differentiates reassortment outcomes between virus populations containing and lacking SI particles even when the total number of particles entering each cell is unchanged. In addition, if instead of holding MOI constant one considers a constant number of infected cells, the particle number required to reach a given level of infection will be higher for parental viruses carrying SI particles. In this situation, the resultant increase in MOI will lead to more co-infection and more reassortment. Thus, SI particles could enhance reassortment by two mechanisms in an infected host. The former mechanism will be more important if the total number of virus particles that can be produced in an infected tissue is limiting, while the latter would be more important if the total number of cells that can be infected is limiting.

The results of Brooke et al. indicate that IAV of diverse strain backgrounds and grown under a range of culture conditions (including in vivo) carry a high proportion of SI particles [18,27]. These results were based on the expression of an incomplete set of viral proteins in cells infected at low multiplicity. Herein, we confirm the presence of SI particles in an additional strain background (Pan/99) and using a distinct methodology (tracking reassortment) to detect SI particles. Our analyses yielded a range of possible values for the probability of a segment being present and, while our results clearly show that P_P_ is not equivalent among the segments, they do not allow more precise definition of P_P_. Nevertheless, our results are consistent with the average P_P_ value for all eight segments estimated by Brooke et al. for an influenza A/Puerto Rico/8/34 (H1N1) virus (average P_P_ of Brooke et al. = 0.781; averages among our 28 best P_P_ settings ranged from 0.65 to 0.78). Coupled with the finding reported herein that SI particles enhance reassortment efficiency, their detection in diverse strain backgrounds suggests that these incomplete virions play a significant role in the evolution of influenza viruses.

Reassortment among variants within a viral population is expected to act like sexual reproduction of cellular organisms in that it allows the combination of multiple adaptive mutations within a single genome, as well as separation of lethal or fitness decreasing changes in one segment from adaptive changes in another segment. In these ways, reassortment is predicted to increase the rate of evolution of a diverse viral population under selection pressure [12,46]. Of course, in the context of a host co-infected with multiple influenza viruses of distinct lineages, reassortment also facilitates genetic exchange that gives rise to large shifts in viral genotype and phenotypes. These instances of genetic shift can very rapidly advance adaptation of an influenza virus to a new environment, including a new host species [4–6,8,12]. For these reasons, the potential for SI particles to increase reassortment efficiency suggests that these virions may accelerate the evolution of IAV.

Defective interfering particles were found experimentally to suppress reassortment relative to that seen with standard virus stocks, but to yield higher levels of reassortment than those predicted for theoretical “perfect” virus stocks that carry only complete genomes. DI particles are similar to SI particles in that they require complementation for infectious progeny production. In addition, DI particles are well known to decrease the production of fully infectious progeny from co-infected cells [17,20,39,42,47]. Thus, DI segments are expected to enhance reassortment by increasing the proportion of productively infected cells that are co-infected, but suppress reassortment by reducing the number of infectious progeny emerging from those cells. Our experimental data obtained with Pan/99-based P3 and P4 viruses suggests that the latter, suppressive, effect of DIs may be most important from a biological standpoint: reassortment levels characteristic of standard virus stocks (that have non-zero baseline levels of SI particles) are lowered by the emergence of DI particles.

Mathematical modeling allowed us to explore whether similar outcomes are expected when the prevalence and/or the potency of interference of the DI segments is varied. The interfering effect of a DI segment arises due to segment-specific competition between a DI and the corresponding standard segment: if a DI PB2 is packaged into a virion, for example, the full length PB2 will not be [45,48]. The accumulation of DI particles over multiple passages indicates that DI segments carrying internal deletions are furthermore more likely to win this competition than are the full-length segments. The mechanism that leads to favoring of influenza virus DI segments over standard segments is not fully resolved, but likely occurs at the level of genome replication and/or packaging and may be related to segment length [44,45,48–50]. Importantly, differing DI segments interfere more or less potently [44]. We found that defective segments that interfere mildly are expected to enhance reassortment relative to the presence of only complete genomes. In contrast, if a DI segment has a strong competitive advantage over the corresponding standard segment, leading to a 10–100 fold reduction in progeny, the presence of DI particles can suppress reassortment. Thus, whether DI particles have a positive or negative impact on reassortment is determined mainly by the potency with which DI segments interfere with infectious progeny production. Our experiments show that, in the context of biological virus populations, the overall impact of all types of DI particles is to suppress reassortment.

Modeling also revealed an additional layer of complexity governing the behavior of DI particles: the presence of SI particles in a virus population can change the expected impact of DI particles on reassortment, presumably by increasing the requirement for complementation. Comparison of experimental data obtained with P3 and P4 viruses to the model suggests, however, that SI particles do not comprise a large fraction of these virus stocks. In this way, the data suggest that, compared to standard segments, DI segments are less likely to be missing. This finding fits well with reports indicating that DI segments are incorporated into virions more efficiently than their full-length counterparts [45,48,51]. Since the behavior of DI particles is complex, it is important to highlight that modeling of segments that compete equally to, or with an advantage over, full-length segments in computational virus populations cannot alone account for the levels of reassortment observed experimentally. Rather, the inclusion in the model of virions that fail to deliver one or more segments to the site of replication is needed to match the experimental data.

We report the effects of SI and DI particles on reassortment in the context of a cell culture model where replication is limited to one cycle. One important difference between this experimental system and infection in an animal host, where IAV will undergo multiple rounds of replication, is in the multiplicity of infection. By tracking reassortment in co-infected guinea pigs, we have seen that MOI increases with viral load in vivo and, at the time of peak shedding, is sufficient to support the production of reassortant viruses at a frequency of about 70% [52,53]. Similar results were obtained whether mixed infection was achieved by intranasal inoculation or through dual transmission events. Thus, MOIs achieved in vivo are sufficiently high to allow complementation of SI or DI particles. The impact of these particles most likely varies with the time after infection, however, since MOI changes as infection spreads in the target tissue. A second important difference between our cell culture model and an animal host is the potential for multiple rounds of replication. DI particles may have a stronger positive effect on reassortment in vivo, since reassortant DI viruses would have the opportunity to be complemented through co-infection in subsequent rounds of replication. Another important consequence of multi-cycle replication in vivo is the potential for rare reassortant viruses to be amplified. If certain reassortant genotypes confer higher fitness than the parental genotypes in the host where they arise, even low overall levels of reassortment can lead to major biological changes.

Our data, and those of Brooke et al., suggest that SI particles outnumber fully infectious particles in a typical IAV population. We have furthermore attributed important biological activities to these particles [18,27]. Nevertheless, the precise nature of SI particles remains unclear. SI particles may lack one or more gene segments due to a failure to package all eight vRNAs during assembly. This possibility is substantiated by lower rates of detection of NA vRNA in a mutant virus population that was shown to have increased SI particle content relative to the wild type strain [54]. A failure to package some segments is not, however, supported by recent fluorescence in situ hybridization data that show a high percentage of virus particles contain eight different viral RNAs [55]. Although not quantitative, electron microscopic analyses of RNPs within IAV virions also show the presence of eight segments arranged in an ordered fashion [56,57]. The possibility that SI particles carry eight fully functional vRNA molecules but fail to deliver one or more to the nucleus is feasible but weakened by FISH analysis of IAV genomes in infected cells, which suggests that the segments remain associated prior to nuclear import [58]. Direct visualization of IAV ribonucleoproteins allowed identification of the polymerase complex bound to the 3’ and 5’ termini of the RNA, but also revealed some segments that did not appear to be associated with a polymerase [59]. Thus, SI particles might carry one or more segments that are not bound by a polymerase complex and are therefore not copied during primary transcription and may be more susceptible to exonuclease activity. While the data presented herein are informative regarding the potential biological implications of SI particles, they do not elucidate the physical nature of these particles, nor narrow down the possibilities listed above.

In summary, our data show that the presence of semi-infectious particles in an influenza virus population increases the potential for genetic diversification through reassortment. This activity of semi-infectious particles is due to an increase in the proportion of productively infected cells that are co-infected, which in turn reflects the need for complementation in order for cells infected with SI particles to produce progeny. Similar to SI particles, the presence of DI particles increases the proportion of infected cells that are co-infected; however, since DI segments inhibit the production of infectious progeny viruses, their overall effect is to decrease rather than increase levels of reassortment relative to those seen with standard virus stocks. We conclude that IAV particles that are not fully infectious may have an important role in influenza virus biology through their effects on reassortment and, in turn, adaptive evolution of the virus.

## Materials and Methods

### Computational methods

#### Simulation setup and MOI variation

We performed computational simulations to monitor levels of infection, co-infection, and reassortment, as a function of a varying multiplicity of infection (MOI). As indicated in Table 3, the MOI was varied dynamically from 1x10^-5^ to 15 particles per cell in (approximately) log-linear steps. This approach minimized stochastic variations at low MOI and increased computational speed for steps at high MOI that do not suffer from stochastic variations.

**Table 3:** Multiplicities of infection evaluated in computational model^1^.

No. virions per type	No. cells	MOI per type
10	1000000	1.000e-5
40	1000000	4.000e-5
158	1000000	1.580e-4
631	1000000	6.310e-4
2512	1000000	2.512e-3
10000	1000000	1.000e-2
39811	1000000	3.981e-2
100000	1000000	1.000e-1
100000	794328	1.259e-1
100000	630957	1.585e-1
100000	501187	1.995e-1
100000	398107	2.512e-1
100000	316228	3.162e-1
100000	251189	3.981e-1
100000	199526	5.012e-1
100000	158489	6.310e-1
100000	125893	7.943e-1
100000	100000	1.000
125893	100000	1.259
158489	100000	1.585
199526	100000	1.995
251189	100000	2.512
316228	100000	3.162
398107	100000	3.981
501187	100000	5.012
630957	100000	6.310
1000000	100000	10
1500000	100000	15

^1^Each row indicates a run. For example, the first run would have 10 virions of type A and 10 virions of type B distributed over 1,000,000 cells, giving an effective MOI of 1x10^-5^ for each type and a total MOI of 2x10^-5^ virions per cell.

#### Infection, co-infection and reassortment of complete genomes

Each virion is randomly assigned to a cell. Based on the number of virions of each type assigned to a cell, the % infection (number of cells infected with any virion type, divided by the number of cells) and % co-infection (number of cells infected by both virion types, divided by the number of cells) is calculated. The expected reassortment level for a cell infected with N_A_ virions of type A and N_B_ virions of type B is:

$$1-{\left({\text{N}}_{\text{A}}/\left({\text{N}}_{\text{A}}+{\text{N}}_{\text{B}}\right)\right)}^{8}-{\left({\text{N}}_{\text{B}}/\left({\text{N}}_{\text{A}}+{\text{N}}_{\text{B}}\right)\right)}^{8}$$

The % reassortment is calculated as an average of the expected reassortment level across all infected cells.

#### Modeling semi-infectious virions

We defined the likelihood for a segment to be present in a virion and copied upon infection as P_P_. Therefore the likelihood that a virion carries and copies all 8 segments is the product of the P_P_ of all eight segments. The model above was modified to account for missing segments as follows. The number of virions that have a given segment, N_P_, was calculated by multiplying the number of virions by the likelihood P_P_ for that segment. From the full set of virions, we selected a random subset of size N_P_, and these virions have the given segment. This process was repeated for all 8 segments, thereby producing a registry of which segments are present for each original virion, after it has been assigned to a cell. This process of assigning segments to virions can give rise to virions with no segments, but this situation is expected to occur at a relevant frequency only with low values of P_P_ for all 8 segments (e.g. with P_P_ = 0.25 for all 8 segments, the chance for a random virion to have 0 segments is 0.75^8^ = 0.100).

For a cell to express HA of a given type, the HA segment of that type needs to be present and, in addition, at least one of each segment NP, PA, PB1 and PB2 needs to be present [1,31,32]. For a cell to be counted as dually HA positive, therefore, both HA of type A and type B are required, as well as at least a single segment of NP, PA, PB1 and PB2.

For a cell to produce progeny, at least one copy of each of the 8 segments is required. Therefore, when semi-infectious virions are present, not all infected and co-infected cells will have progeny. For cells producing progeny, the expected reassortant frequency is calculated as follows:

$$\begin{array}{l} \text{expected reassortment}=1–\{N_{NA}{}_{{}_{A}}/(N_{NA}{}_{{}_{A}}+N_{NA}{}_{{}_{B}})*N_{HA}{}_{{}_{A}}/(N_{HA}{}_{{}_{A}}+N_{HA}{}_{{}_{B}})*N_{M}{}_{{}_{A}}/(N_{M}{}_{{}_{A}}+N_{M}{}_{{}_{B}})*N_{NS}{}_{{}_{A}}/(N_{NS}{}_{{}_{A}}+N_{NS}{}_{{}_{B}})*N_{NP}{}_{{}_{A}}/(N_{NP}{}_{{}_{A}}+N_{NP}{}_{{}_{B}})\\ {}*N_{PA}{}_{{}_{A}}/(N_{PA}{}_{{}_{A}}+N_{PA}{}_{{}_{B}})*N_{PB1}{}_{{}_{A}}/(N_{PB1}{}_{{}_{A}}+N_{PB1}{}_{{}_{B}})*N_{PB2}{}_{{}_{A}}/(N_{PB2}{}_{{}_{A}}+N_{PB2}{}_{{}_{B}})\}-\{N_{NA}{}_{{}_{B}}/(N_{NA}{}_{{}_{A}}+N_{NA}{}_{{}_{B}})*N_{HA}{}_{{}_{B}}/(N_{HA}{}_{{}_{A}}+N_{HA}{}_{{}_{B}})\\ \;*N_{M}{}_{{}_{B}}/\left(N_{M}{}_{{}_{A}}+N_{M}{}_{{}_{B}}\right)*N_{NS}{}_{{}_{B}}/\left(N_{NS}{}_{{}_{A}}+N_{NS}{}_{{}_{B}}\right)*N_{NP}{}_{{}_{B}}/\left(N_{NP}{}_{{}_{A}}+N_{NP}{}_{{}_{B}}\right)*N_{PA}{}_{{}_{B}}/\left(N_{PA}{}_{{}_{A}}+N_{PA}{}_{{}_{B}}\right)*N_{PB1}{}_{{}_{B}}/\left(N_{PB1}{}_{{}_{A}}+N_{PB1}{}_{{}_{B}}\right)*N_{PB2}{}_{{}_{B}}/\left(N_{PB2}{}_{{}_{A}}+N_{PB2}{}_{{}_{B}}\right)\} \end{array}$$

Where N_YZ_ represents the number of segment Y present for virion type Z (A or B). The expected % reassortment is calculated as the average of the expected reassortant frequencies across all cells producing progeny.

To evaluate which set of P_P_ values corresponded best to the measured data, P_P_ was varied between 0.25 and 1.0 in increments of 0.25. We tested all possible combinations, taking into account the redundancy of PB2, PB1, PA and NP as well as that of NA, M and NS. These redundancies arise because the polymerase and nucleoprotein components are each required for an infected cell to be HA positive, and removing any one of these segments therefore has the same impact on HA positivity. Conversely, the presence of NA, M and NS are not required for a cell to be HA positive and, again, removing any one of this group has the same impact on HA positivity. The absence of any segment impacts reassortment, since all eight segments are needed to generate infectious progeny. Thus, for example, P_P_ = 0.25 for PB2 gives the same reassortment and HA positivity outcomes as P_P_ = 0.25 for PB1. In total, we evaluated the 2800 different combinations of P_P_ values by comparing them to the measured data.

From the resulting simulations, we interpolated the HA positive, dually HA positive and reassortment values to get 20 values between any original set of two simulation values, in the graphs of % HA positive versus % dually HA positive, % HA positive versus reassortment, and % dually HA positive versus reassortment. From these three now high-resolution data curves, we calculated for each P_P_ value the sum of distances of the experimental data to the interpolated simulation curves (sum of errors). The optimal P_P_ settings, which would have the smallest sum of distances between the data and the graphs, was then determined by taking the sum of the three sums of errors from the three graphs. The optimal settings were: P_P_(PB2): 0.25; P_P_(PB1): 0.5; P_P_(PA): 0.75; P_P_(HA): 0.75; P_P_(NP): 1; P_P_(NA): 1; P_P_(M): 1; P_P_(NS): 1; both virus types A and B had the same setting per segment.

#### Modeling UV treatment of virions

The probability of UV treatment introducing a lesion in a given segment is directly related to their length [60]. For a given UV dose U_D_ the proportion present for a specific segment was modified as follows from P_P_ to P_P,UV_:

$$P_{P,UV}=\;P_{P}*\;\left(1\;–\;U_{D}*\;L/13623\right)$$

Where L is the length of the segment (2341 for PB2 and PB1, 2233 for PA, 1762 for HA, 1566 for NP, 1466 for NA, 1027 for M, 887 for NS). A UV dose of 2 would on average hit two positions per genome with this model.

This value P_P,UV_ was then used instead of P_P_ to determine which virions had which segments present, as described in “Modeling semi-infectious virions”. Using the set of P_P_ values determined above, we then varied the UV dose between U_D_ 0 and 5 in steps of 0.05. The sum of errors was then calculated, as described for “determining the P_P_ value”. The minimum of the sum of the three error values was found for U_D_ 2.0 hits per genome, which following the Poisson distribution corresponds to an expected 13.5% of genomes being unaffected by the UV treatment.

#### Testing the role of MOI in determining the relationship between P_P_ and reassortment

To better understand the mechanisms underlying changes in reassortment levels seen with changes in P_P_, we modeled reassortment and co-infection levels as functions of P_P._ For these simulations, we varied the P_P_ from 0.02 to 1 in steps of 0.02 and performed 20 repeats. Using 10^5^ cells, we calculated the number of virions of type A and B needed to achieve a given MOI, e.g. 3000 virions of type A and 3000 of type B for an MOI of 0.03. The average % reassortment was calculated across all progeny-producing cells (i.e. those cells with at least one copy for each of the 8 segments), or all co-infected, progeny-producing cells, as indicated. Of the 10^5^ cells, those that were producing progeny were either categorized as co-infected (if containing segments of both type A and B), or singly infected (if only containing segments of one type).

#### Modeling defective-interfering virions

We defined the probability that a segment is not a DI as the probability intact, P_I_. DI particles were then added to the model as follows. After determining which virions have which segments present (based on P_P_), the probability that a segment is intact, P_I_, was used to determine the number of virions that have a segment present and intact, according to N_PI_ = N_P_*P_I_. From the virions with the segment present, we selected a random subset of size N_PI_, and these virions have the given segment present and intact. All the other segments that are present, but not intact, represent defective-interfering segments. We modeled defective interfering segments for PA, PB1 and PB2 of virus types A and B [37,38,44], and allowed all six segments to have different probabilities of being intact. The resulting simulation produced a registry of which segments are present and intact for each original virion, after it has been assigned to a cell.

For cells producing progeny, the expected reassortant frequency is calculated per the formula above, where N_YZ_ represents the number of segment Y present *and intact* for virion type Z (A or B). However, because the DI segments reduce the number of infectious progeny produced, the expected % reassortment is calculated as a *weighted* average of the expected reassortant frequencies across all cells producing progeny, where the weighting factor is calculated as:

$$weightingfactor=\;DIX^{\#DI}$$

That is, the factor DIX decreases the progeny produced per DI particle present, as #DI represents the number of DI segments present within that cell.

We evaluated, for three settings of DIX (0.01, 0.1 and 0.45), a series of 125 settings of P_I_, where the PB2, PB1 and PA P_I_ were varied independently from 0.2 to 1.0 in increments of 0.2, while the P_I_ of all other segments and the P_P_ for all segments was set to 1. Additionally, we made a model where DIX was varied from 0.05 to 0.5 in increments of 0.05, the experimentally measured P_I_ parameters were used (Table 2), and P_P_ was set to the settings found optimal above (P_P_(PB2): 0.25; P_P_(PB1): 0.5; P_P_(PA): 0.75; P_P_(HA): 0.75; P_P_(NP): 1; P_P_(NA): 1; P_P_(M): 1; P_P_(NS): 1; same settings for A and B). We then repeated this model with P_P_ = 1 for all segments.

Finally, we evaluated for three settings of DIX (0.01, 0.1 and 0.45), a series of 125 settings of P_I_, where the PB2, PB1 and PA P_I_ were varied independently from 0.2 to 1.0 in increments of 0.2, four different settings of P_P_: i) all segments 1; ii) all segments 0.8; iii) all segments 1, except PB2, which is 0.1; iv) the best P_P_ settings found previously (0.25,0.5,0.75,0.75,1,1,1,1).

### Experimental methods

#### Ethics statement

Embryonated hens’ eggs obtained from HyLine International were incubated for 9–11 days and then used to propagate influenza viruses. Animal ethics board approval was not required for this work because the eggs were not allowed to hatch and therefore do not constitute live animals.

#### Viruses and cells

Madin-Darby Canine Kidney (MDCK) cells were obtained from the ATCC and maintained in minimum essential medium (MEM) supplemented with 10% FBS and penicillin-streptomycin. Infections were carried out in serum-free MEM supplemented with 3% Bovine Serum Albumin and penicillin-streptomycin. Recombinant influenza A/Panama/2007/1999 (H3N2) [rPan/99wt-His] and rPan/99var2-HAtag viruses were described previously [30,61]. Briefly, these viruses were generated by reverse genetics and propagated in 9–11 day old embryonated hens’ eggs for one or two passages. rPan/99var2-HAtag virus contains the following silent mutations relative to rPan/99wt-His virus (nucleotide numbering is from the 5’ end of the cRNA): NS C329T, C335T, and A341G; M C413T, C415G and A418C; NA C418G, T421A and A424C; NP C537T, T538A and C539G; HA T308C, C311A, C314T, A464T, C467G and T470A; PA G603A, T604A and C605G; PB1 C364T, T348G and A351G; and PB2 T621C, T622A and C623G. In addition, the two viruses differ in the epitope tags inserted into the HA protein after the signal peptide [30,62], with the wt virus encoding a His tag and the var virus encoding an HA tag. Collectively, these mutations were shown not to attenuate the growth of rPan/99var2-HAtag virus relative to rPan/99wt-His virus in MDCK cells [30].

#### UV irradiation of virus stocks

Pyrimidine dimers were introduced randomly into the rPan/99wt-HIS and rPan/99var2-HAtag viral genomes by partial UV inactivation. Each virus stock was diluted to 1x10^8^ PFU/ml in PBS and separately placed into a 6-well culture dish (340ul/well). Each culture dish was placed approximately 30cm from a 254nm, 8W, UV light source and exposed for 35 seconds.

#### Co-infection at a range of MOIs with untreated and UV treated viruses

Untreated and UV treated rPan/99wt-HIS and rPan/99var2-HAtag virus inocula were prepared in parallel, from the same virus stocks. As indicated above, each virus stock was diluted to 1x10^8^ PFU/ml in PBS. One aliquot of each virus was UV treated and a second aliquot of each virus was “mock treated” (placed in a culture dish but not put under the UV lamp). Untreated rPan/99wt-HIS and rPan/99var2-HAtag virus stocks were then mixed in a 1:1 ratio. Similarly, UV-treated rPan/99wt-HIS and rPan/99var2-HAtag virus stocks were mixed in a 1:1 ratio. Each virus mixture was then diluted with PBS to the appropriate titer for inoculation at MOI 10, 6, 3, 1, 0.6, 0.3 and 0.1 PFU/cell of each virus. These MOIs were based on the viral titers before UV treatment.

Infections were performed in 6-well dishes seeded with 4x10^5^ MDCK cells per well on the previous day. For inoculation, dishes were placed on ice, growth medium removed, and monolayers washed with PBS three times. Each well was inoculated with 250ul volume, on ice, and cells were incubated at 4°C for one hour to allow virus attachment. The inoculum was aspirated and any unattached virus removed by washing the monolayers three times with PBS. After the addition of virus medium (MEM supplemented with 3% BSA and Penicillin/Streptomycin), cells were transferred to 33°C. At 3 h post-infection, virus medium was removed from the monolayers and replaced with medium containing NH_4_Cl (MEM supplemented with Penicillin/Streptomycin, 20mM NH_4_Cl, 50mM HEPES buffer and 0.1% BSA) and returned to 33°C. At 12 h post-infection, supernatant was collected and stored at -80°C for subsequent genotyping of released virus. MDCK-infected cells were harvested and prepared for flow cytometry (see below).

#### Generation of virus stocks with high levels of DI particles

Virus stocks with abundant DI particles were generated by serial passage in MDCK cells at high MOI. For the first passage, Pan/99wt-His and Pan/99var-HAtag viruses were used to infect MDCK cells in a T75 flask at MOI = 5 PFU/cell. Passages 2–5 were performed blindly, using 2 ml undiluted cell culture supernatant from the previous passage as inoculum in each T75 flask. Incubations at each passage were performed at 33°C for ~48 h. To gauge particle:PFU ratio at each passage, PFU titers were determined in MDCK cells according to standard techniques and RNA was extracted from 140 cell culture supernatant (QiaAmp Viral RNA Kit), subject to reverse transcription with Univ.F(A) primer and Maxima RT (Fermentas), and analyzed by quantitative PCR using primers specific for NS, M and NA segments with BioRad SsoFast Evagreen supermix. PCR was run in triplicate and median Ct values were used in further analyses. RNA copy numbers of P1 –P5 (Px) viruses relative to the corresponding progenitor stock (P0wt or P0var) were calculated for each segment according to the equation 2^(-Ct_Px_) / 2^(-Ct_P0_). The average result for all three segments was then calculated to give the relative RNA copy number for each passage. PFU titers of P1 –P5 stocks were also normalized to those of the corresponding P0 stock. Finally, RNA:PFU ratios shown in Fig 10 were calculated as relative RNA copy number divided by relative PFU titer.

#### Measurement of relative DI particle content

We used an RT qPCR assay to determine the proportion of gene segments with large internal deletions in the P3 and P4 stocks, relative to our standard stocks (referred to as “P0”). Total RNA was extracted from 140 ul samples of each virus stock using the QiaAmp Viral RNA Kit (Qiagen) and reverse transcribed using the Univ.F(A) primer and Maxima RT, according to the manufacturer’s instructions. Viral cDNAs were then subjected to qPCR using SsoFast Evagreen supermix (BioRad) and a panel of 16 primer pairs. The panel comprised two primer pairs for each segment: the first set bound within 150 nucleotides of the 3’ terminus and the second set bound internally, in a region typically deleted within DI segments [37,38] (primer sequences available upon request). The procedure was carried out three times on different days to give three biological replicates and each assay included three technical replicates. The proportion of viral gene segments that were intact (P_I_), relative to P0 stocks, was calculated according to the following formula, where “Px” refers to P3 or P4 viruses, “internal” indicates Ct values obtained with primers binding at an internal site and “terminal” indicates Ct values obtained with primers binding within 150 nt of the 3’ end of the vRNA.

PI=[2^(− Ct internal)/2^ (− Ct internal)]PX/[2^(− Ct internal)/2^ (− Ct internal)]P0

This calculation was performed separately for each segment. The median Ct value among the three technical replicates was used for the calculation. The median P_I_ values determined from three independent experiments are reported in Table 2.

### Co-infection at a range of MOIs with P0, P3 and P4 virus stocks

The RNA:PFU ratios were higher for rPan/99wt-HIS virus than for rPan/99var-HA virus at each passage (Fig 10). Appropriate volumes of P0 rPan/99wt-HIS virus were therefore spiked into both P3wt and P4wt viruses to yield stocks with RNA:PFU ratios equivalent to their P3var and P4var counterparts. P3wt and P3var virus stocks were then mixed in a 1:1 ratio and P4wt and P4var virus stocks were similarly mixed in a 1:1 ratio. One-to-one mixtures of P0wt and P0var viruses were also prepared. Each virus mixture was diluted with PBS to the appropriate titer for inoculation at MOI 3, 1, 0.3, 0.1, 0.03 and 0.01 PFU/cell of each virus. MOI 3 was not carried out for the P4 viruses due to insufficient titers. PFU values refer to those of the P0 viruses. The amount of P3 and P4 viruses used in each infection was based on the RNA:PFU ratios. Thus, equivalent units of RNA were used for P0, P3 and P4 infections and the amounts of RNA used corresponded to 3–0.1 PFU/cell of the P0 viruses. Inoculation of MDCK cells was performed as described above for untreated and UV treated viruses, except that medium was not changed at 3 h post-infection to introduce NH_4_Cl. At 16 hours post-infection, supernatant was collected and stored at -80°C for subsequent genotyping of released virus. MDCK-infected cells were harvested and prepared for flow cytometry (see below).

#### Measurement of % infection, % co-infection and % reassortment

Infection and co-infection levels were determined as described previously [30]. Briefly, flow cytometry using commercial antibodies specific for a His tag (Qiagen Penta-HIS Alexa Fluor 647 conjugate, Mouse IgG1 [item# 35370]) and an HA tag (Sigma Aldrich monoclonal anti-HA-FITC, Clone HA-7 [item# H7411]) allowed enumeration of Pan/99wt-His and Pan/99var2-HA virus-infected cells, respectively. Singly and doubly infected cell populations were gated manually using uninfected cells treated with both anti-His and anti-HA antibodies as negative controls.

To calculate % reassortment, virus genotypes were determined by high resolution melt analysis essentially as described previously [30,52]. Briefly, the following steps were performed. 1) Plaque isolates were obtained by plaque assay of MDCK cell supernatants. 2) RNA was extracted from agar plugs using the Zymogen 96 Viral RNA kit, according to the manufacturer’s protocol except that 40 ul water was used for elution. 3) Twelve microliters of RNA was reverse transcribed from an IAV-specific “universal” primer (Univ.F(A): 5’ GGCCAGCAAAAGCAGG) using Maxima reverse transcriptase (Fermentas) according to the manufacturer’s instructions. 4) cDNA was used as template in qPCR reactions with the appropriate primers [30] and Precision Melt Supermix (BioRad) in wells of a white, thin wall, 384 well plate (BioRad). qPCR and melt analyses were carried out in a CFX384 Real-Time PCR Detection System, as per the instructions provided with the Precision Melt Supermix. Data were analysed using Precision Melt Analysis software (BioRad). Viruses were scored as reassortant if the genome comprised a mixture of wt and var gene segments in any proportion. Occasionally, one or more gene segments gave an ambiguous result in the melt analysis. In these cases, virus isolates were excluded from the analysis if >1 segment could not be typed. Where only one segment could not be assigned as wt or var, the genotype was recorded based on the remaining seven segments.

## Supporting Information

S1 Fig A model in which incomplete virions carry defective non-interfering segments, rather than lacking a segment, does not yield a good fit with experimental data. To test whether observed reassortment levels (plotted with black circles) could be explained by the presence in co-infecting virus populations of incomplete particles carrying segment(s) with lethal point mutations (i.e. defective non-interfering segments), the model was modified as follows. All virus A and virus B segments were assigned P_P_ = 1.0. Since lethal mutations are expected to occur in any segment, P_I_ was varied for all eight segments over a range of 0.25 to 1.0 in increments of 0.25. All 2800 possible combinations of P_I_ were tested, taking into account the redundancy of PB2, PB1, PA and NP as well as that of NA, M and NS. Defective segments were assigned a DIX value of 0.5, so that they have equal probability of being incorporated into progeny virions as do standard segments. A sum of errors analysis was then performed to identify the P_I_ settings that yielded the best fit with the data. The results obtained with the top 1% of settings are plotted here with colored lines showing relationships between % HA positive cells and % dually HA positive cells in (A) and % HA positive cells and % reassortment in (B). The 28 lines are colored from best (blue) to worst (red) fit. Low P_I_ values for all eight segments were required to achieve the fits shown (e.g. for the best fitting setting, P_I_ was 0.25, 0.25, 0.25, 0.5, 0.25, 0.25, 0.25, 0.25 for segments 1–8, respectively). Under these conditions, reassortment levels are subject to a high degree of stochastic variation because very few cells are producing virus. Thus, although the colored lines in (B) show overlap with the data points to some extent, these results are spurious good fits that can arise from stochastic variability. A true good fit would resemble the neat smooth curves seen for example in Fig 5. In addition, the stochasticity apparent in the modeled results presented here is not seen in the experimental data. In contrast, experimental data obtained with P4 DI-rich virus stocks do show stochastic variation as predicted by the model. Finally, we note that the P_I_ settings found to best fit the data predict particle to PFU ratios on the order of 10,000:1, which are not biologically plausible. Taking these considerations into account, we concluded that modeling of defective non-interfering particles rather than semi-infectious particles does not offer a good solution for the experimental data.
(EPS)

S2 Fig Results obtained with P_P_ = 0.9 for DI-containing viruses were consistent with those observed following P3 and P4 virus co-infections. We found that P_P_ values that gave a good match between the model and data obtained with standard virus stocks did not work well for DI-rich virus stocks. Higher P_P_ values were needed to achieve a fit with reassortment data obtained with the P3 and P4 viruses. Here we show that P_P_ set to 0.9 for all eight segments yields a reasonable match between the model and P3 or P4 datasets. A and B) Relationship between % HA positive cells and % dually HA positive cells. C and D) Relationship between % HA positive cells and % reassortment. P_I_ parameters measured for the P3 (panels A and C) and P4 (panels B and D) virus stocks were input into the model. DIX was varied from 0.05 to 0.5 in increments of 0.05 and is shown with a color scale in each panel, where blue represents DIX = 0.05 and orange is DIX = 0.5. A dashed line representing modelled results in the absence of DI particles (P_I_ = 1.0) is plotted as a reference.
(EPS)

S3 Fig Theoretical interplay among P_P_, P_I_ and DIX in determining reassortment outcomes. To capture the inter-relationships among P_P_, P_I_ and DIX in determining reassortment levels, we evaluated in the model four representative P_P_ settings (shown above each column for segments 1 through 8), three disparate DIX values (shown to the left of each row), and 125 different P_I_ settings in which values for PB2, PB1 and PA were varied independently from 0.2–1.0 in increments of 0.2 (shown within each panel with colored lines). The color assigned to each of the 125 P_I_ settings reflects the product of P_I(PB2)_, P_I(PB1)_ and P_I(PA)_, with the highest product (0.8) in red and the lowest product (0.008) in blue. The line corresponding to P_I_ = 1.0 for all segments is shown in black. These results indicate that there is a complex interplay among P_P_, P_I_ and DIX in determining reassortment levels. Patterns emerge, however, with a comparison among the panels. A larger effect on reassortment outcome is seen with a change in P_P_ (moving across the figure), compared to a change in DIX (moving down the figure) or in P_I_ (which is varied within each panel). Indeed, the overall effect of varying P_I_ is found to depend strongly on the settings used for P_P_.
(EPS)
